# Don’t Follow the Smoke—Listening to the Tobacco Experiences and Attitudes of Urban Aboriginal Adolescents in the Study of Environment on Aboriginal Resilience and Child Health (SEARCH)

**DOI:** 10.3390/ijerph20054587

**Published:** 2023-03-04

**Authors:** Christina L. Heris, Mandy Cutmore, Catherine Chamberlain, Natalie Smith, Victor Simpson, Simone Sherriff, Darryl Wright, Kym Slater, Sandra Eades

**Affiliations:** 1 National Centre for Aboriginal and Torres Strait Islander Wellbeing Research, Australian National University, Acton, Canberra, ACT 2601, Australia; 2 Centre for Epidemiology and Biostatistics, University of Melbourne, Parkville, Melbourne, VIC 3000, Australia; 3 Sax Institute, Glebe, Sydney, NSW 2037, Australia; 4 Indigenous Health Equity Unit, University of Melbourne, Parkville, Melbourne, VIC 3000, Australia; 5 Riverina Medical and Dental Aboriginal Corporation, Wagga Wagga, NSW 2650, Australia; 6 Tharawal Aboriginal Corporation, Airds, Sydney, NSW 2560, Australia

**Keywords:** Aboriginal and Torres Strait Islander Health, adolescents, tobacco, smoking prevention, health promotion, community program

## Abstract

Preventing smoking among young Aboriginal people is important for reducing health inequities. Multiple factors were associated with adolescent smoking in the SEARCH baseline survey (2009–12) and discussed in a follow-up qualitative study that aimed to inform prevention programs. Twelve yarning circles were facilitated by Aboriginal research staff at two NSW sites in 2019 with 32 existing SEARCH participants aged 12–28 (17 female, 15 male). Open discussion around tobacco was followed by a card sorting activity, prioritising risk and protective factors and program ideas. The age of initiation varied by generation. Older participants had established smoking in their early adolescence, whereas the current younger teens had little exposure. Some smoking commenced around high school (from Year 7), and social smoking increased at age 18. Mental and physical health, smoke-free spaces and strong connections to family, community and culture promoted non-smoking. The key themes were (1) drawing strength from culture and community; (2) how the smoking environment shapes attitudes and intentions; (3) non-smoking as a sign of good physical, social and emotional wellbeing; and (4) the importance of individual empowerment and engagement for being smoke-free. Programs promoting good mental health and strengthening cultural and community connections were identified as a priority for prevention.

## 1. Introduction

Tobacco use continues to be a major cause of preventable ill health for Aboriginal and Torres Strait Islander peoples accounting for half of all deaths in people aged 45 years and older, and is the largest contributing modifiable risk factor to the gap in disease burden between Aboriginal and Torres Strait Islander people and non-Indigenous Australians [1,2]. While there have been significant declines in daily smoking prevalence among Aboriginal and Torres Strait Islander people in recent years, it remains almost three times higher than in the non-Indigenous population [3,4,5,6,7,8]. Promisingly, two recent studies have found an increasing proportion of young Aboriginal and Torres Strait Islander people have never smoked [9,10]. As most regular smoking begins in adolescence and young adulthood, preventing youth uptake will continue to drive down smoking prevalence among Aboriginal and Torres Strait Islander people and reduce the gap in health outcomes [9,11,12,13]. Thus, understanding the factors related to smoking and non-smoking among young Aboriginal and Torres Strait Islander people is important for designing effective prevention programs.

Generally, the drivers of smoking initiation and tobacco use among Aboriginal and Torres Strait Islander people are similar to those of other populations. These include normalisation and exposure to peer or family smoking, concurrent use with alcohol and cannabis, as well as life stress and poor mental health [13,14]. Smoking is strongly correlated to the social determinants of health, with greater prevalence among those experiencing social disadvantage, living in remote areas, and those with lower levels of education engagement or employment opportunities [11,14,15,16]. The continuing disparity in smoking prevalence between Aboriginal and Torres Strait Islander people and non-Indigenous Australians reflects the overrepresentation of Aboriginal and Torres Strait Islander people in these key determinants and how, through ongoing colonisation processes, these structural inequalities have become entrenched [11,17]. This is compounded by experiences of racism and discrimination, intergenerational trauma, dislocation and disconnection, and the historical manipulation of the Aboriginal population through the provision of tobacco in rations [14,17,18,19]. Conversely, positive cultural determinants of health are an important counterbalance, promoting resilience and wellbeing [20]. Tobacco prevention programs that are comprehensive and also address the structural inequalities and the underlying determinants of smoking are thought to be most effective, particularly through Aboriginal Community Controlled Health Services (ACCHS) [15,17,20,21].

The Study of Environment on Aboriginal Resilience and Child Health (SEARCH) is a community-controlled cohort study of Aboriginal children, young people and their carers from four Aboriginal Community Controlled Health Services (ACCHS) in urban and regional New South Wales (NSW) (in this paper, unless referring to specific data sources of both Aboriginal and Torres Strait Islander populations, the term Aboriginal, rather than Aboriginal and Torres Strait Islander, is used as this is a NSW-based study where this is the accepted terminology). It provides a unique insight into the individual, social, environmental and cultural factors associated with the smoking experience of young Aboriginal people in urban areas. Cross-sectional baseline (Phase 1—2008-12) findings from self-reported responses from Aboriginal adolescents (12–17 years) and their carers identified multiple factors that may be associated with adolescent tobacco use, and have the potential to inform policy and community-level programs [22]. These include the findings that young people were significantly more likely to have never smoked regularly if they had good mental health, their mother as their primary caregiver, good family relationships, stable housing, had never used alcohol, were not sexually active and had no criminal justice interactions. Other factors that are likely important but did not show a statistically significant relationship were related to age, school engagement, environmental tobacco smoke exposure, cannabis use, and community connections. A relationship between tobacco use and alcohol, cannabis and e-cigarettes has also been demonstrated in other samples of Aboriginal and Torres Strait Islander adolescents [23,24,25].

Other factors identified through a previous systematic review [26] which have a relationship to smoking but were unclear in the SEARCH analysis included participation in sport and recreation, cultural factors, experiences of racism, stressful life events, socioeconomic indicators and gender. This review included findings from some earlier qualitative studies conducted with young Aboriginal and Torres Strait Islander people in other Australian jurisdictions [27,28].

This study aimed to understand and explore the smoking and non-smoking experiences, attitudes and beliefs of Aboriginal adolescents aged 12 and above in urban and regional settings in NSW, and to triangulate the cross-sectional SEARCH survey findings to develop recommendations for future, contextually relevant, community-based prevention programs. Specifically, this study sought to explore, interpret, confirm and explain the quantitative findings with two generations of SEARCH adolescents (Phase 1 and 2 participants), and through discussion, identify key factors to be leveraged in future community-led tobacco prevention interventions.

## 2. Materials and Methods

### 2.1. Qualitative Approach

This is the second stage of an explanatory sequential mixed methods [29] research project serving to validate and extend the initial cross-sectional survey findings [22]. This mixed methods approach is useful for understanding adolescent tobacco use [30], triangulating the initial survey findings to develop an understanding of the study participants’ experiences, behaviours, attitudes, beliefs, and motivations. The study uses a phenomenological approach [31] with a yarning methodology [32,33,34], and is reported here in line with the COREQ guidelines [35].

Phenomenological methods such as interviews and group discussions are commonly used to understand, describe and interpret behaviours and the meanings attached to experiences, and are particularly useful for exploring perceptions, attitudes and beliefs [31]. Group discussions also allow for observation of participant interaction and exploration of differences in response [36]. For young people in particular, small group discussions have the added advantage of allowing people to respond to the experiences of others. Here, individual and group discussions are used to provide an in-depth and flexible exploration of the issues identified in the SEARCH quantitative analysis [22].

Our discussions were underpinned by an Aboriginal research method, yarning. Yarning allows for culturally safe and more relaxed, interactive, collaborative, two-way conversation in Indigenous settings, reducing the formality and power dynamics of traditional research interviews [32,33,34]. This study integrated both social and research topic yarning [33,34]. The Aboriginal Research Officer (MC) began with social yarning, sharing his background and experiences, building familiarity and a relationship with participants over morning/afternoon tea prior to commencing the research topic yarns. The non-Aboriginal researcher (CH) also shared her background and experience, and the participants introduced themselves. The research topic yarns were guided by the participants’ stories and were two-way discussions with MC also sharing experiences and responding to questions himself.

### 2.2. Researcher Characteristics

ACCHS staff and Aboriginal researchers have led the design and conduct of this study. Co-authors SS and CC guided the research design, one a SEARCH Aboriginal Research Officer familiar with the study, its partners and participants (SS), the other a senior Aboriginal academic (CC) experienced in qualitative research methods. The senior author (SE), also a senior Aboriginal academic, provided leadership and guidance throughout. Another SEARCH Aboriginal Research Officer (MC) led the yarns, supported by CH (a non-Aboriginal researcher) and in some sessions, with other Aboriginal ACCHS staff (NS, VS, KS).

### 2.3. Setting

Fieldwork was conducted at two ACCHS partner sites in NSW, one regional and one metropolitan. Staff identified suitable spaces in which participants would feel comfortable and safe. At both sites, these were recreational community spaces, separated from dedicated health service buildings and with an informal, relaxed lounge atmosphere. Morning/afternoon tea was provided.

### 2.4. Ethics

AHMRC (Aboriginal Health and Medical Research Council of NSW) approval was granted by amendment to the SEARCH study ethics (568/06) and registered with University of Melbourne HREC (1953833). CEO approval from both sites was received. Additional informed consent protocols were followed with each participant receiving a participant information sheet and consent form (PICF). SEARCH ROs sought caregiver consent for 12–17-year-old participants in advance, with the underage adolescents also providing their own assent when attending. Key points from the PCIF were reinforced prior to each session.

### 2.5. Sample & Recruitment

ACCHS/SEARCH staff recruited 32 existing study participants (aged 12–28 years) of any smoking experience who had either participated in SEARCH Phase 1 (between 2008-12) or Phase 2 (from 2014) when aged between 12–17 years. Participants were primarily recruited in small groups of 3–4 friends or family within kinships groups. Group composition was determined by ACCHS staff based on their local understanding of relationships and dynamics that would increase participant comfort and familiarity to facilitate discussion. Recruitment continued at Site 2 until saturation was reached, and participants shared similar perspectives. All participants received a Gift Card ($50 value) and morning/afternoon tea. The gift cards and sharing of food acknowledged the value of their contribution and helped build a reciprocal relationship.

### 2.6. Data Collection Methods

Fieldwork was completed in March and June 2019 across the two sites, with a total of 12 yarning circles (9 group and 3 individual yarns) of approximately 60 minutes each. Each began with introductions as outlined above, followed by a broad, general discussion about health and specific tobacco knowledge, attitudes and experiences. Participants then completed an interactive card sorting activity of 45 potential risk and protective factors drawn from the SEARCH survey items [22,37] and Australian and international tobacco literature [26]. Participants were given a share of the cards to organise into protective or risk factors for smoking, highlighting any that were unclear or could go both ways. A detailed discussion of the key selected factors followed, with participants encouraged to share different opinions on card placement. The sessions concluded with a discussion of potential prevention program designs (covering objectives, audience, settings, timing and key components).

The primary purpose of the card sorting activity was to validate [30] the findings of the SEARCH Phase 1 survey in an interactive way, allowing participant stories to explain the identified statistical associations [22]. Other relevant factors identified in a systematic review were also added, and thus the card sorting activity triangulates both the SEARCH survey findings and international evidence [22,26].

This is an effective method, particularly among young people, vulnerable populations or when discussing sensitive topics [38]. Not only is the research more engaging and dynamic [38]; participants considered a range of factors that they may not have discussed unprompted, and did so through a third-party projective technique [38] of discussing a hypothetical ‘other’ person similar to them. Less vocal, shy participants were able to contribute by adding their cards to the overall arrangement without needing to share their own personal experiences if they did not want to.

#### 2.6.1. Instruments

A short discussion guide was used and revised between Site 1 and Site 2, removing redundant prompts duplicating the discussion. The guides, cards and fieldwork photos are included in Supplementary Files S1–S3. All sessions were recorded using a digital audio recorder, with MC reading out the card groupings for the recording.

#### 2.6.2. Data Processing

The fieldwork team debriefed at the end of each interview. Audio files were transcribed by a third-party contractor with no connection to participants. CH reviewed the transcripts against the recordings for immersion and accuracy, removing identifying information. Redacted transcripts were sent to MC and the other SEARCH ROs for checking. A multi-stage coding process was undertaken in NVivo 12 by CH. Site 1 coding was completed prior to Site 2 fieldwork.

#### 2.6.3. Data Analysis

Thematic analysis was used as described by Braun and Clarke and Green et al. [39,40,41], taking a phenomenological approach, and guided by the Theory of Triadic Influence [42,43], an ecological model of individual, social and environmental influences on smoking that was referenced in the first phase of this study [22]. Data familiarisation and immersion was carried out as described above, followed by line-by-line coding in NVIVO to key categories (open codes); the data were then organised into clustered concepts (axial codes) to identify initial subthemes in order to develop a thematic map of interconnections between concepts and subthemes and then generate the overarching themes. Both inductive (unprompted discussion) and deductive coding were used, and updated iteratively with some reclassification. Nodes for the topics listed on the cards were pre-set and actively sought in the data (deductive). See Supplementary File S4 for the final coding structure.

The relationship between the risk and protective factors as identified/described by participants was initially mapped by CH and organised into four overarching categories, loosely guided by the Theory of Triadic Influence. From this, four key analytic themes were developed in collaboration. CC reviewed a visual summary of risk and protective factors and independently organised them into similar themes. Concurrently, MC reviewed a full summary of the analysis with the major themes, sub-themes and illustrative participant quotes, and by teleconference confirmed that they reflected the yarns. Finally, CH compared these agreed findings to those from the Phase 1 quantitative findings [22].

#### 2.6.4. Member Checking/Validation of Themes

The identification of key themes, findings and recommendations was completed in collaboration with several Aboriginal co-authors (CC, SE, MC, SS, NS). A short summary of the major themes and key findings was presented to the ACCHS partners for feedback. COVID-19 restrictions prevented the research team from presenting findings to participants at a local event, but these were presented to staff via Zoom. A written summary was also made available to participants via the ACCHS. Feedback from the ACCHS staff and participants was incorporated where received. AHMRC and the ACCHS partners provided approval for publication.

## 3. Results

### 3.1. Results Overview

Overall, 32 participants were recruited for twelve yarning sessions across the two sites, with a diverse range of ages and smoking experiences (Table 1).

Four key themes were generated from the analysis: (1) drawing strength from culture and community; (2) how the smoking environment shapes attitudes and intentions; (3) non-smoking as a sign of good physical, social and emotional wellbeing; and (4) the importance of individual empowerment and engagement in being smoke-free.

We briefly discuss general concepts of health and wellbeing and personal experiences of smoking/non-smoking, then detail influences on tobacco use under the four key themes (summarised in Table 2), and conclude with community prevention program recommendations. A detailed summary of participant quotes is presented in Supplementary File S5.

#### 3.1.1. General Concepts of Health and Wellbeing

Tobacco use was not top of mind for many participants when they were asked about healthy versus unhealthy behaviours. Half the groups spontaneously mentioned smoking and was almost exclusively listed by non-smokers as a behaviour to avoid. However, when prompted, smoking was considered one of the unhealthiest behaviours (after ‘hard’ illicit drugs). For all groups, ‘healthy’ was about diet and exercise. Social and emotional wellbeing, poor mental health and some specific diseases (diabetes, cancer) were also mentioned. Some also mentioned broader determinants of health such as education, employment, personal safety (including domestic violence), living in a hygienic environment, social and cultural connections, as well as broader concepts of life balance, motivation and agency to look after themselves and pursue their interests.

#### 3.1.2. Personal Exposure and Experiences of Smoking/Non-Smoking

Participants had a range of tobacco experience (Table 1), including regular smokers, occasional ‘social’ smokers, and several who had only experimented. Around half had never smoked.

Those who had tried or taken up smoking as a teenager reported starting between 10–15 years old, with friends or family (often cousins and siblings) facilitating first attempts. Some only began around age 18–21, when they started working, going out to bars, and were legally able to buy cigarettes.

Smoking intensity varied. For the ‘social smokers’, this was dependent on when they were socialising/drinking, and might be ‘1–2 cigarettes every second weekend’. For the current regular smokers, intensity ranged from 8–10 cigarettes a day for some up to 30–40 for others, and varied depending on whether the individual was having a “bad day”.

All non-smokers were firm in their intentions not to start smoking in the future. The occasional smokers did not explicitly say they wanted to stop, and although they acknowledged it was not good for them, they rationalised that they did not smoke often. All current regular smokers intended to quit in the future or were actively cutting down. Although they listed a range of barriers to quitting, they passionately described what kind of cessation support they would value.

I think I only smoke due to my peace, that’s like my peace. You know. My little getaway from my little one and partner and yeah. And I think I smoke…I really do, I just can’t find myself to quit right now. When I become more stable, when I get my own house that I know I’m not going to get kicked out of. And when I get the kids settled, and like I can start focusing on my health again. I really want to quit, I just can’t right now. I think once I get more stable, and it’d be easier for me to quit.

[11.F1]

### 3.2. Key Smoking/Non-Smoking Themes

**Figure 1:**
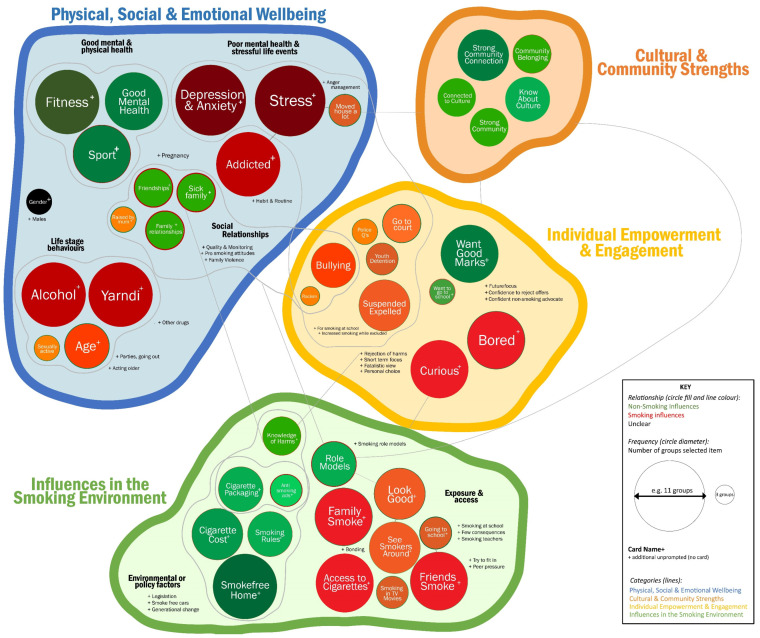
Concept map of the card sorting activity. This figure provides a visual summary of the grouping of key factors by theme and in relation to whether they were non-smoking (green) influences or smoking influences (red). The size of the circle reflects the number of groups that selected the card. A large-scale version is provided in Supplementary File S6.

The relationship between the key factors under the four overarching themes (Table 2) is illustrated in Figure 1 and described in the following sections. A large-scale version of Figure 1 is provided in Supplementary File S6. This concept map illustrates the grouping of topics raised in the card sorting activity (both those pre-determined on the cards and those raised spontaneously) to form the key themes. The topics are grouped under the themes and subthemes, with lines indicating where participants identified relationships across themes (for example, having a family member with a smoking-related illness (within Theme 3) also relates to knowledge of the harms of tobacco use (under Theme 2)). The more commonly a group nominated a particular card or issue as important for smoking prevention, the larger the circle. In the diagram, protective factors are indicated with a green colour, and risk factors with a reddish tone. In cases in which the card was primarily identified as either a risk or protective factor but an alternative view was raised, the alternate-coloured ring is present (e.g., role models were identified as being protective against smoking, except when they were smokers; this is represented as a green circle with a red ring).

#### 3.2.1. Theme 1. Drawing Strength from Culture and Community

##### Community, Cultural Connections and Role Models

Having *strong community* and *cultural connections* were important protective factors that were highlighted by all groups as central to supporting young people to stay smoke-free or quit smoking. Community belonging and the non-smoking values of the community were also important. Strong *role models***,** as they are related to *community connection***,** included wanting to be a good role model and contribute to the community as a leader. However, the smoking behaviour of role models also relates to Theme 2.

I reckon, just like if you’re someone that’s deeply connected with your culture, it gives you like um, I don’t know, just like a sense of belonging, and it just makes you not want to smoke because you’re more interested in, in your culture. But, like, so could be either studying, it could be working with the community. It could be um, studying your culture. Like yeah, and just, I feel that being connected to your culture, it does um make you want to smoke less, just because of wanting to fit in with the, the rest of your community and stuff like that.

[11.F2]

Role models. Just don’t be a follower, be a leader. And then strong community, ah, connection to my community. So, keep everyone in the community strong, try and quit smoking. Be healthy. Yeah, live longer.

[4.M1]

#### 3.2.2. Theme 2. How the Smoking Environment Shapes Attitudes and Intentions

Participants attitudes to smoking and their own experiences were strongly shaped by the environment and the behaviours of those around them. *Normalisation of smoking* through the influence of having *friends* and *family who smoked***,** which created *access to cigarettes*, as well as *seeing smokers around* the community or even *on TV/in movies* were all seen to contribute to smoking.

##### Role Models

While *strong role models* had the potential to be an important and positive influence (Theme 1), this is mediated by whether they themselves smoke and what behaviours they model.

Like role model, like it’s in there, but I feel like it can kind of be wrong, cos if your role model smokes, you’re going to kind of want to be like him.

[7.F1]

##### Friends Who Smoke and Smoking at School

Almost all participants felt that *smoking friends* were a particularly important influence for initiation. Some reported experiencing overt *peer pressure* to try smoking, or doing so to fit in and belong to the group. For others, it was simply *curiosity*. Some reported starting with *social smoking* at parties or on a work break, which then evolved to regular smoking. Current smokers who started socially, expressed *regret* that their lives had moved on from those social connections but they were still smoking.

Yeah, it’s going through your mind “what are they going to think of me if I say no”.

[2.F2]

Yeah, I’ve tried cigarettes but I don’t smoke, occasionally. Like when I’m around parties and all that, it’s kind of a social thing where you feel pressured to smoke, in front of everyone. And so obviously, I just usually do it at parties I think.

[10.F1]

[RO] Do you think that your main reason for starting was just because of your friends? Yep?

And now I’ve got no friends I’ve only got me and my two kids

[2.F1]

[RO] Kids. Yep.

It’s nothing to be proud about now.

[2.F1]

But, when I became an adult and started working and stuff, and started hanging round people that did smoke, I did end up picking it up. So I shouldn’t even have started. I’ve only been smoking for about four or five years, but since I have been smoking, I’ve gotten pretty bad at it.

[11.F2]

[I1] Um, how’d you feel after your first cigarette?

I started choking a lot. Like, I couldn’t really handle it, that was then, and then I started a lot. But, um, yeah. I started smoking probably only socially. So I’d go and just have a yarn with the girls, I’d have a smoke. And then soon it started me going out by myself to smoke. And then I just started going again and again, and I just never stopped.

[11.F2]

Most non-smokers had very low visibility of smoking among their peers, with their close friends also being non-smokers. While some were aware others their age smoked, they perceived this negatively. Non-smokers held strongly negative attitudes to smoking. They felt smoking peers were doing so for their self-image: *trying to be cool, act older and fit in*. Other attitudes ranged from smoking being disgusting or ‘gross’ (particularly reinforced by those who had a bad personal experience trialling smoking); a bad habit; wrong (particularly if the smoker is young); silly or stupid; a waste of money; and shameful, embarrassing and uncool.

Everyone just thinks like they’re wannabees. But, then they think to themselves “oh, we’re top shit” and all that…They think that they’re like seventeen, but they’re like twelve, fifteen

[7.F1]

For some urban participants, even among non-smokers, there was very high visibility of smoking at their schools, and these participants could easily identify key locations within school grounds at which smokers congregate. One school had a very *permissive smoking environment*, and participants estimated that at least half of all students smoked with little consequence, as some teachers also smoked in sight of students. Smoking in the toilets during class time was reportedly common.

The teacher smokes, so…

[8.F2]

It can really put an influence on the kids

[8.F1]

But, the teachers go outside the school gate and, or some of them, or some of them wait until after school.

[8.M1]

Positive, reverse peer pressure was described by one participant who was worried about losing his friends and about what they would think of him if he started smoking. Although pressure from peers to smoke was reported by many, some felt they could confidently say no and avoid personal exposure. Some non-smokers also reported how smoking created tension in relationships when encouraging friends to stop. These points also relate to self-efficacy and confidence under Theme 4.

##### Smoking among Family Members

The majority of participants did have *family members* who smoked, and this visibility was perceived to directly contribute to smoking initiation through observation and curiosity or direct encouragement as well as *normalisation* of smoking. For the older participants, this normalisation created an environment in which smoking was more a collective action, and a behaviour that was transferred generationally as opposed to an individual behavioural choice, with smoking taken up almost unconsciously in this social context. Two participants reported their mothers had first quit when they fell pregnant and continued to be smoke-free.

My mum’s a big smoker too. She started smoking when she was about twelve. Same with my brother. His father, he started smoking when he was about twelve. And my sister, she started smoking at a young age too. But, because I had health problems, I didn’t start smoking till later in life.

[11.F2]

Yeah, Mum’s always been a smoker, since a young age, and so was her mother, well now me and my brother and sisters smoke, and my brother’s kids smoke.

[11.F2]

But, my son, he’s only two, and his nan like came down from [Town2] and she just, she was staying at my house and she kept going out the back smoking. And after she left, he’d get up and be like “mum I want a moke”. That’s what he said…That’s what he said. And I was like “what?”. And then he’s like “I want a moke”. But, see, cos he noticed her smoking. Cos like nobody else in the house smokes, but seeing her kept, keep going outside, and then he probably thought he’d be able to go outside if he smoked. Like that’s, it shocked me when he said that.

[1.F1]

Whether family members smoked or not did not automatically correspond to the adolescent’s own smoking behaviours. One participant lived in a smoke-free household and reported sneaking out to smoke. For some, smoking family members were motivation for their own smoke-free choices, but others reflected with some irony that although they had been strong non-smoking advocates in their early years, social influences had led them to take it up themselves. These were occasional ‘social’ smokers who felt there was a difference between their smoking and their families’ regular smoking.

[I1] What were your thoughts though when you seen your mum and that smoking?

Oh, I thought well “like, why do you smoke? Don’t do that, I don’t want you to die” or something, you know what I mean? And then now I’m doing it. Only when I drink though but still, I don’t reckon I should do it, cos, it’s not a healthy thing.

[4.M1]

[I1] How does that make you feel, like having a night out with cigarettes?

I feel normal. It’s gross. They are, they’re gross. Um, most of my family smokes, not any more. My Nan used to smoke, she got cancer from it. Um, I used to hide her smokes from her. She used to get really angry. It was really funny. You know, it was a good thing. I used to, cos she had cancer, she wasn’t supposed to smoke. So. Yeah.

[10.F1]

[I1] So knowing that Nan had cancer, what made, what made you want to smoke?

Mostly just people around me. I don’t do it all the time, you know, just mostly when I’m around people.

[10.F1]

##### Smoke-Free Homes and Second-Hand Smoke Exposure

Having a *smoke-free home* and *anti-smoking rules* (parents’ expectations or designated smoke-free spaces) were identified as key to preventing smoking. Several participants lived in households with very strong anti-smoking expectations, which translated to their own smoke-free intentions.

No participants reported living in homes where smoking was allowed inside, and many said visitors were respectful of this expectation and would ask permission to go outside and smoke. Some also noted that ‘outside’ was understood to mean far away from the door and out by the fence.

While there have been important changes in the home environment, reducing *second-hand smoke* (SHS) exposure away from home was still a concern for some. One young mother felt that not all community members were aware or concerned about the harms of SHS, and that did not think to move away from a pram in public places, but that she was unable to say anything. One young participant reported feeling upset by being involuntarily exposed to smoking by their peers.

##### Knowledge of Harms and Impacts from Smoking

*Knowledge* of the harms of smoking was high among all participants. They described several physical health impacts including death, cancer, damage to lungs or breathing problems as well as being unhealthy in general, impact on fitness and more cosmetic impacts to teeth, smell, and skin. Many had first-hand experience of the harms of smoking, with family members who were sick or had passed away from smoking-related illnesses, including SHS exposure.

*Financial and social costs* due to high cigarette prices and loss of future opportunities or time with family were also mentioned, alongside harms to others from SHS and being a burden to family. Occasional ‘social’ smokers perceived different levels of harm from lower smoking intensity, and felt that was an important distinction between them and regular smokers.

Although knowledge of the harms of smoking was high, information sources were unclear or uncommon. Some groups reported learning about them at school; however, overall, this seemed infrequent, and a lower order issue behind mental health. Awareness of campaigns was low, with most reporting that they had seen fewer advertisements in recent years other than posters at the AMS. However, some were able to recall and describe specific ads (‘Terry’, ‘Everybody knows’, Nicorette) and calls to action (‘Call Quitline’, ‘get the app’), and one participant said that these ads were the main reason they did not smoke.

Many were able to refer to specific graphic health warnings on packets (and to even describe the “Brian” execution). However, some questioned the impact of warning labels and campaigns, as many people still smoked. This relates to a recurring sub-theme that knowledge of harms was not enough to prevent smoking, particularly when smoking facilitated bonding or social cohesion. This was not discussed as a positive perception of smoking, but more as an explanation for why participants had started or continued to smoke socially, despite knowing the harms of smoking and holding negative views on it.

I think that’s the main reason I don’t do it. Cos you know, you see ads and that on TV. Um, yes. My biggest turnoff. Lung cancer and that’s a big thing. Don’t want to do it. Cos you put your family through a lot and that.

[5.M1]

Yeah, I see ads and that, like quit smoking, call smoke line, or help line or whatever it is. I don’t know what it is. I seen it on ads.

[4.M1]

[AHW] Do you see much though?

No I hardly see stuff about smoking to be honest. Only thing I see is all the stuff from the packets of people like dying and yeah. That stuff. That’s the main thing I see on the smokes.

[4.M1]

[I1] What do you think when you see those?

It’s horrible.

[4.M1]

[I1] Makes you not want to smoke.

Yeah, it does. It does. I don’t want to end up like that. Know what I mean? I don’t want anyone to end up like that.

[4.M1]

##### Historical Knowledge and Awareness of Policy or Legislation Change

Younger participants had little awareness of the changes in tobacco control over the past two decades, and of how the formerly pro-smoking environment had contributed to the long-term dependency they saw in their families. Older participants who smoked were acutely aware of these changes over time, and reflected that when they started, it was common to be around smoking inside cars, homes, restaurants and bars. They expressed regret at starting, noting that it was becoming harder to smoke, with increasing prices and restrictions making it ‘not worth it’.

You had more freedom back then. And the smokes were cheaper back then. They were cheaper and you were allowed to smoke in more places. You know, there weren’t that many rules, but now there’s so many rules around it, and they’re so expensive, and it’s just, it’s starting to feel like it’s not worth it anymore, but it’s really hard to quit.

[11.F2]

There was also very little knowledge of the *historical* introduction of tobacco to Aboriginal people. MC provided some tobacco education as part of the yarning circles on the history of use in Australia; this was of particular interest to younger participants, and demonstrated an opportunity to incorporate this history into formal tobacco education programs.

So, the laws we have now and that, do you, like wish they were there when you were little?

[9.F2]

[I1] Yeah, definitely. Yes I do, yeah… But, these are things that I sit back actually, and think on, if all these things that are part of your generation now, the laws, and restrictions, if that was in place when I was younger, then yeah. Cos, you know, when I look back on it now, um, majority of my Elders have all passed away from cancer, from cigarettes. So, very preventable diseases. Um, if the laws had changed many, many years ago, then a lot of my Elders would still be alive today. I know that for sure. And the fact that cigarettes and alcohol was an introduced thing to our people you know, makes it even, um, makes it more upsetting in a way. There was no need for our people to have these influences given to us.

##### Vaping

Only four groups spontaneously mentioned *vaping (e-cigarettes)* and three had direct experience or exposure. One had observed vapers in public but generally dismissed it as embarrassing behaviour. One participant asked if it was for quitting and described a friend who switched from smoking and continues to vape at a similar intensity.

A group of urban secondary students reported high levels of vaping among their peers, particularly the use of ‘JUULs’ and ‘vape pens’ that look like USB sticks and pens. They reported the devices being brought to school and used in the classroom, describing large clouds of vapor, attempts to blow smoke rings and a range of flavours. Generally, there was little or no knowledge of their contents or potential harms; the participants stated that their peers believed that they contained no nicotine (just water or air), and that vaping was less harmful than smoking.

Vaping’s stupid. What’s the point of that? [Mimes cloud of smoke]

[6.F1]

[I2] Maybe they think they look like a magician or something.

Yeah, like, they reckon they’re mad. They make themselves shame. So shame. Dumbest thing ever created…I see someone what an idiot, how stupid is that. How stupid is it to just stand there, inhale some smoke and blow it out? “Oh because it tastes nice.” It’s so stupid.

[6.F1]

Vaping. Lot of that around now.

[9.M2]

And Shisha as well… A lot of people at night go do shisha.

[9.M4]

There’s a lot of that [vaping] going around.

[9.F2]

Yeah, they all try to blow circles.

[9.M4]

They even do it in class.

[9.M3]

The teacher will be writing on the board and they’ll go and then put it in their bag. It’s funny.

[9.M2]

#### 3.2.3. Theme 3. Non-Smoking as a Sign of Good Physical, Social and Emotional Wellbeing

##### Substance Use

*Alcohol and other drug use* were seen as particularly important for adolescent smoking initiation. Many reported only smoking when they drank, or that initiation occurs at the same time as drinking begins, when going to parties and licensed venues; established smokers reported that their smoking increased when they drank. Mixing tobacco with cannabis was understood to be common practice which then contributed to nicotine addiction.

##### Sport and Fitness

Participation in *sport and fitness* was seen as protective against smoking, through keeping fit and active more broadly, maintaining a healthy mindset, not being bored and drifting into other activities (relates to Theme 4), as well as not wanting to risk impairing performance.

And if you’re, for sport, if you’re in a rep team, or if you want to be big in a particular sport, sporting, ah, smoking. Won’t help you. It’ll just make you go down track, and it’ll just be bad for you, in anywhere you want to be.

[7.M1]

##### Mental Health and Stress

For many, smoking was a symptom of greater concerns, and it was acknowledged that good health and social and emotional wellbeing was important for being a non-smoker. One participant said he would see newly commenced smoking in friends as an indicator of what else may be going on in their lives, underlying drivers at home or in their relationships, and an opportunity to check-in.

I’d worry about what’s going on with their family and stuff. Start asking questions and that…there’s always a reason they’re doing it. Starting smoking. There’s always a reason.

[5.M1]

Poor mental health and stress were seen as key drivers of smoking. Stress was discussed widely, and while there were general descriptions of “stress”, many specific factors were seen to contribute to stress which then increased the risk of smoking. These included having seriously ill family members and grief if they had passed away, moving house frequently, experiences of bullying, being in trouble at school or with the police, and being expelled or going to youth detention (which relates to Theme 4).

It was rare for smoking to be discussed positively, except for use as a stress management tool, a form of self-medication and an opportunity for a time-out. Particularly for participants that had small children and had made their homes smoke-free, smoking provided an opportunity to step outside and have a break, as their children knew to stay away when someone was smoking. Smoking was also described as a way of practicing mindfulness, as an activity that forced them to slow down and focus on breathing.

A bit of both. Like I know the kids can’t come near me if I’m having a smoke. Cos I go outside and smoke, like “you stay inside, I’m out here smoking, leave me alone”. That’s why, five minutes for me to sit down and what I’ve been told, to have a smoke, you literally physically have to calm down because if you’re running, or if you’re walking around, you can’t smoke as well. Because your lungs are working to help you breathe. So, you’ve got to sit down, relax so then you can inhale the cigarette. It is forcing you to sit still and to relax, just so you can smoke a cigarette. That’s, yeah, so to have a cigarette you need to relax yourself, and yeah. I use that a lot.

[11.F2]

Cos I’ve moved houses quite a lot too, since I was about eighteen. And I felt like it did worry me sometimes cos I didn’t know where I was go…I was couch hopping. I moved probably ten, fifteen times in the last couple of years. And right now, I’m in a transitional house, so I’m going to be moving again in a few weeks. Maybe, or it could be a month, or it could be two. I don’t know. So, it’s always the, the fear of not knowing where I’m going to be able to set up a permanent house for me and the kids, yeah, cos we haven’t had stability, we’ve moved, I’ve had the kids for about a year and a half now, and I’ve moved house three times. And we’ve got to move again, and again, and it’s just like, it stresses me out knowing that we can’t unpack all our stuff, just because we know that we’re going to be packing up again in a few months. And the kids, it’s not fair for the kids. They don’t get a chance to set up their rooms and they don’t feel like they’ve got a stable home. Hmm. So that does stress me out as well…That’s why I need my smokes.

[11.F2]

##### Nicotine addiction

Many tied *nicotine addiction* to stress, in that smoking was used initially to manage stress and over time developed into an addiction.

It comes under stress and, as well, because once you smoke, it, that’s a way that it could make your stress go away. And once you have that, like…

[7.M1]

Once you start smoking…

[7.M2]

Yeah, you get heaps addicted to it because it takes away the stress.

[7.M1]

It could be like depressed. Like, feel like you harm yourself or whatever. And you need a smoke to calm you down or something. For example like yarndi. And then same thing, like if you’re stressed out, like going through court, family members in hospital crook. Just want to smoke, try and relax yourself. And then addicted, since you started, you’re just addicted. Ever since you started.

[4.M1]

##### Family Illness

While a *serious illness in the family* was seen as a stress that could be managed by smoking, if it was a smoking-related illness, non-smokers found this reinforced their decisions not to smoke. However, for some established smokers, stress outweighed knowledge of the harms of smoking (related to Theme 2).

If you have a family member that’s sick and you see like, the pain and the stage that they’re going through. You feel like upset because you don’t want them to be like in that, in that stage, and it will help you not want to be like them, in the bed.

[7.M1]

Like yes it would turn me off smoking, cos obviously all the bad stuff instilled into your body and stuff, but it would also make me want to smoke, just because of the stress factor involved. So, my family member’s dying, like there’s nothing I can do, it’s just it’s gotta stress me out and it’s my family’s all stressing. Like, we’re going to bury somebody, they’re dying. Um, it would just like put my stress levels right up. So I’d be smoking because of the stress, but I’d also be thinking like I need to quit because look what it’s done. I know that it’s doing more harm than good. Yeah. But, just at that time, you feel like you just need to have a smoke just to catch your breath, catch your thoughts, just stop for a second.

[11.F2]

##### Relationships

The role of family relationships and friendships was unclear. These relationships were perceived to be a potential source of strength for preventing smoking or supporting quitting, but very much depended on the quality of the relationship and whether they were supportive of non-smoking (relates to Theme 2).

…if you feeling stressed your family can help you instead of smoking. Cos when people are stressed they smoke, or if other people are stressed, then their family can help them.

[7.M2]

Cos if you have a strong family, they’re behind you, you know, make you quit. Or you have a bad family that doesn’t give a shit, you know, they just keep lending you.

[9.M3]

#### 3.2.4. Theme 4. Importance of Individual Empowerment and Engagement for Being Smoke-Free

##### Disengagement, Boredom and Rejection of Harms

While many believed there was no particular reason behind smoking, and that it was simply a *personal choice*, different experiences and attitudes informed those choices. Young people who were *bored and disengaged* (which includes getting in trouble at school or with the police) were seen to be at higher risk of smoking, being *short-term focused* and interested in cultivating the *rebellious* or ‘cool smoker’ image, and *rejecting the harms* of smoking by rationalising infrequent, low levels of consumption while they were young (related to Theme 2).

I think it’s also to do with trying to look cool. Um, I think you shouldn’t have to wreck your body to try and look cool.

[9.F1]

They’re all doing it because they think it won’t happen to them, till like a couple of years…Yeah, they’re not worried about the long term, they worry about short term…they’re like you know, “I’ll quit after school”. When school finishes, they’ll quit, but they just keep going.

[9.M2]

##### Empowerment, Engagement and Self-Efficacy

Young people who were more positively *engaged* with their community and culture (Theme 1) and future focused, including at school and in thinking about their life post school, were more *empowered* to resist smoking even when in high smoking environments (Theme 2). They displayed the *self-efficacy* to navigate smoking environments, and were very comfortable and confident in rejecting offers, removing themselves, publicly advocating against smoking and advising others not to smoke. For them, their long-term career and family goals were directly tied to their short-term smoking behaviours.

[I1] And you’ll be a non-smoker?

Yeah, I got a future career I’ve got to focus on. Can’t do that shit.

[12.F2]

At parties. Do it a lot at parties when they’re drinking. They decide to smoke. I don’t really like it. I just walk away from them. Cos I don’t really like even seeing them doing that…I like being different. And I like, you have to be your own person, you don’t have to follow other people. I’d rather be a leader than a follower.

[5.M1]

[I1] What makes you think “oh, I don’t want to do that”?

Just your future.

[3.M3]

Ask them what their dreams are. And then if they say whatever, then you say you’re not going to get it by smoking.

[3.M1]

### 3.3. Designing a Prevention Program

Participants provided their thoughts on potential programs to reduce smoking in their communities in relation to core objectives, target audience, settings, key features and timing.

#### 3.3.1. Objectives

Participants agreed that there is a need for general smoking prevention that provides information and education to young people prior to initiation. However, it is also important to encourage quitting attempts and offer cessation support to young people who are already smoking regularly and have developed a nicotine dependence. Broader healthy lifestyle and skills programs that include a focus on nutrition, mental health and stress management, other substance use, and managing peer pressure (for smoking and other behaviours) and that promote a general positive life and future focus would be valued.

#### 3.3.2. Target Audience

There is a need for tobacco prevention to reach young people across the total period of adolescence and young adulthood. The early teenage years/when starting high school (aged 12–13 years) are a particularly important period for experimentation. Thus, it was felt that education should begin in primary school (students in Year 5 or 6 and from age 8–10 years) so that students are primed with knowledge of the harms of smoking before they entered high school, at which time access and exposure increases. Others noted that this needed to be sustained throughout high school years, as information provided at age 12 was likely forgotten by the age of 16–17 years. Further, with turning 18 (another key period for initiation), it was thought that programs needed to continue to reach people into their twenties.

While some felt there was an opportunity for a whole of community program that reached smokers of all ages, others felt there had been a focus on supporting Elders to quit, and it was now time to focus on the youth. It was noted that selection of age groups must be managed carefully, as some young people would not want to participate with adults (approximately 26 and over), whereas others felt it was important to allow younger children in because they often feel excluded. There was no real distinction made by sex or gender.

#### 3.3.3. Settings

A range of settings and approaches were discussed. More advertising and social marketing was suggested, including the development of a TV show featuring young people talking about their own smoking experiences.

There was an expectation that health professionals would continue to raise quitting with patients, but greater follow-up may be needed, as people may resist the first attempts to discuss smoking.

Schools were expected to be a place in which students could learn about the harms of smoking, and for those attending high-prevalence schools, it was strongly felt that it was important to address these harms in that setting. Sporting clubs were also an avenue to offer advice/education, and Aboriginal community spaces were seen as a key setting for all programs, particularly those with a broader health and wellbeing focus.

#### 3.3.4. Features

Younger participants were not supportive of any program that was delivered as a straight health education exercise. They felt attendance would need to be compulsory and was unlikely to impact existing smokers. Participants requested hands-on interactive learning (such as a model diseased lung they could handle) and personal stories from role models, Elders and cancer survivors who could share the realities of smoking. It was important for the program to be fun and interesting.

Be like, if they can get something like chemicals here, like I don’t know. Like show like how smoking works. Like you know how they have that one lung and the other lung, and how you press it and how slow the air released out of the smoked lung?

[7.M2]

I reckon we should get some Elders that’s like grew up smoking and quit…Sit around and tell you how much, tell the young ‘uns you know, how it ruined them, and how they felt to inspire the little kids not to do it.

[9.M2]

Young mothers requested a women’s group, and were interested in opportunities to quit as a group while also making new friends, being supported socially and engaged in regular cultural activities (such as painting, cooking, jewellery making).

Because there are a lot of young mums around here, that smoke, well not just mums, single people too. Um, but yeah. Because there’s been a bit of talk about wanting, I’ve asked a few girls about the women’s group, and a lot of them want the women’s group back. I do. I’ve been saying that for years.

[11.F2]

Key incentives to promote attendance and participation were the provision of good food, and some Koori-designed apparel (such as a t-shirt) only available to program participants.

Well, like T-shirts are a very big deal around here. So if there was a deadly designed T-shirt at the end of it, that you get to keep, or maybe um, like a bag or something, that’s covered in Koori designs, that’d, that’d be a real eye catcher.

[11.F2]

A range of diversional activities were suggested that would help keep young people to be active and engaged with community and subsequently help protect them against smoking and other risk behaviours. These included providing computer access, game nights, movie nights, a disco, a music festival, sports, and non-team based physical activities (dancing, yoga, swimming). There was also demand for a large range of cultural activities: art classes, camps, cooking classes with traditional recipes, cultural awareness through dancing, games, music (including learning how to make and play a didge), and Aboriginal women’s, men’s and youth groups. There was a desire and an expectation that a key part of any program would be improving wellbeing through greater cultural and community connections.

#### 3.3.5. Timing

Suggestions for timing varied by the type of program suggested. For regular youth diversion activities, a weekly/twice weekly schedule was recommended. It was expected that quit support groups would run for at least nine months to allow people to relapse and re-join as needed, and to be supported in the longer term to stay quit and build relationships. For regular youth-targeted community events that might include a small health messaging component, it was also suggested that these could occur at the end of each term.

At least nine [months], yeah. Cos if I want to quit smoking, I know my behaviour’s going to change and I want her there with me, cos if she’s quitting too, she’s going to want me, and it’s just going to be like, we know we’ve got another couple of months to go together, and like. So by the time we full on quit, you know, it’d be good to make new friends too. Like there are other people who I don’t really talk to, but I’m pretty sure if we like go through this program, and meet up, and we’ll have something in common about quitting smoking … Just something constant over the next few weeks that we know is going to be in place. So like I’m not going to go cold turkey and then start smoking in a month or two, you know what I mean?

[11.F2]

## 4. Discussion

In this study, smoking attitudes and experiences varied by age. Older regular smokers established smoking in early adolescence, whereas most teenage participants had little to no personal experience or exposure (depending on their school environment). Early high school was a key period for experimentation. ‘Social smoking’ increased around 16–18 years. Most had family members who smoked, but none reported living in houses where smoking was allowed inside. Mental health, stress, addiction, other smokers and substance use were key risk factors. A strong connection to community and culture, physical and mental wellbeing, social relationships and non-smoking environments were protective. Hands-on, interactive programs with personal stories and age-relevant cessation support were recommended, with broader diversional, community-based cultural activities. Young people who were strong anti-smoking advocates and were able to confidently reject offers of cigarettes even in high-prevalence smoking environments were engaged at school, socially and in sports, and were future-focused and well supported by family.

Four major themes were developed:

1. Drawing strength from culture and community;
2. How the smoking environment shapes attitudes and intentions;
3. Non-smoking as a sign of good physical, social and emotional wellbeing;
4. The importance of individual empowerment and engagement in being smoke-free.

### 4.1. Comparison to SEARCH Survey

The discussions mostly confirmed the quantitative findings from the SEARCH baseline survey [22], in particular the association between smoking and poor mental health and alcohol use, and provided context for weaker or unclear relationships. Due to the small sample size in the survey, yarndi use was not significantly related to smoking; however, in this study, there was strong understanding of the relationship. While it was acknowledged that not everyone who smoked tobacco used yarndi, yarndi users tended to mix in tobacco (with some aware of such users identifying as non-smokers). This mixing has been demonstrated elsewhere [44].

Stress was a key reason for smoking in this study. Although the survey measure of ‘stressful life events’ was not significant, several individual measures were also identified as important by participants. These included being in trouble at school, and the unintended consequence of school exclusions facilitating smoking and increasing disengagement. Participants agreed that family arguments could create a stressful home environment that increased risk of smoking (and the inverse, that good family relationships were important for non-smoking, particularly if parents were non-smokers). Sick family members also contributed to stress that some managed with smoking, but this increased the salience of the harms of smoking for others. Moving house frequently increased instability and life stress, which may also contribute to smoking. Although criminal justice interactions showed a strong association in the quantitative analysis and participants broadly agreed this would contribute to life stress, it was not an obvious relationship in the qualitative study. Some younger non-smokers avoided smoking to reduce the risk of being picked up by police. Others noted that you could no longer smoke in prison/detention.

Although not a significant survey finding, smoking was understood to increase with age; however, it was noted that it could still begin in the early years, as a way for younger people to try to act older. While participants were perplexed by the reported strong relationship between smoking and being sexually active in the survey, some felt this also related to a life stage of experimentation and acting older.

Although not statistically significant in the survey, non-smoking parents and smoke-free homes were identified by participants as clear non-smoking influences. Similarly, a strong sense of community or knowledge of Aboriginal culture and history were not significantly associated with smoking behaviour in the survey results, but factors related to community and cultural connection were identified in this study as vital for supporting young people to be smoke-free and make quit attempts (the exception being where community members were smoking and encouraged uptake). While many of our participants felt this connection was important for prevention, few stated that this was their own reason for being smoke-free, but that it was an asset they would like to access more.

Satisfaction with available leisure activities was thought to be important, as many felt boredom was a key risk factor and that keeping active and engaged was a protective factor. Higher household income was inconclusive, as it could both increase the affordability of cigarettes and support other activities for avoiding boredom and disengagement.

### 4.2. Comparison to International Evidence

A systematic review [26] of influences on Indigenous adolescent tobacco use in Australia, New Zealand, Canada and the United States informed the card sorting activity. This study confirmed key risk factors of smoking among family, friends and the broader environment, stress, poor mental health and boredom, and protective factors of smoke-free homes, physical activity and strong relationships. Many studies in this review referenced academic engagement and performance as protective against smoking, but school attendance was a risk factor if there was a culture of smoking at school. This was strongly confirmed by our participants, particularly in the urban groups, with school engagement considered protective as part of future career aspirations; however, for those in very pro-smoking school environments, school engagement did not equate to non-smoking. The review also found that cultural connectedness had the potential to be protective, but the available evidence was very limited and included very few studies. This is reinforced by the null findings from the SEARCH survey and our qualitative findings.

### 4.3. Comparison to Other Qualitative Studies

Our findings support previous qualitative work with Aboriginal adolescents and adults in other settings [27,28,45,46,47]. The normalisation of smoking in high-prevalence family and community environments is a particularly strong theme here and in other studies [27,28,45,46] that has seen smoking integrated into Aboriginal identity in some south-eastern communities wherein connection to culture has been disrupted [46]. The way in which smoking as a shared experience and the sharing of cigarettes facilitates relationships and maintains kinship bonds [27,45] was also raised in our study, as was the important influence of smoking behaviours modelled by family leading to the intergenerational transfer of smoking [45], and the converse protective effect of parents’ non-smoking values (even when smokers) reinforcing smoke-free environments [27]. Similarly, the very strong social influence of peers was emphasised in our study and was a key finding for multiple studies, describing bonding, belonging, peer pressure and a desire to be ‘cool’; it was also found that this could be counterbalanced by non-smoking peers [27,28,46,47].

A high level of awareness of the harms of smoking has been reported by others; this included the perception that harms were not relevant to short-term ‘social smokers’ who did not identify as ‘real smokers’ [28,47], despite evidence that ‘lighter’ levels of smoking are harmful [48]. Similar to our participants, one study observed the occurrence of a transition from young, non-smoking advocates to smokers when their behaviour was socially reinforced [46], and observed the strong relationship to socialising with alcohol and cannabis, boredom and curiosity [27,46,47]. Stress was also a major recurring theme in other studies [28,46,47] with participants similarly describing smoking to calm down, relax and cope with stressful events [28].

In line with program preferences in our study and findings on future-focused aspirational non-smokers, Passey et al. emphasised culture and identity as a source of empowerment, but also that marginalisation of young Aboriginal people contributed to a short-term focus and smoking risk [46]. The recommendations made in their study were to maintain engagement with school and sport, and create opportunities for social support and connection to happen without smoking [46]. Some further shared findings were the need to provide young people with the tools to manage stress and the relevant support to quit [28], and to continue to de-normalise smoking in adolescents’ social environments and communities [27,28,47].

### 4.4. Recommendations for Programs

1. *Prevention programs must build cultural and community connections, empower young people and draw on community strengths.* This essential approach is operationalised nationally in both the Australian Government’s ‘My Life My Lead report and Implementation Plan for the National Aboriginal and Torres Strait Islander Health Plan 2013–2023′ (the Implementation Plan) [20,49]. Three out of four Implementation Plan goals for adolescent and youth health are tobacco related, with key strategies for success including pride in culture, support to make healthy choices and involving young people in the design of programs.
2. *Young community members must lead the development of prevention programs to build youth engagement and empowerment.* Many study participants demonstrated they were eager and ready for such an opportunity. A potential model would see centralised decision making by an independent youth committee supported by and embedded within existing community-controlled organisational structures, providing leadership opportunities and enhancing self-determination while ensuring the program’s appeal and relevance.
3. *Interactive education about the harms of tobacco use is needed and should incorporate storytelling alongside youth-specific cessation support.* It is important to counter the perception of safer, ‘light’ levels of smoking among ‘occasional’ or ‘social smokers’, as even lower levels of consumption increases cardiovascular disease risk [48]. The low knowledge of, but clear interest in, Australia’s history of tobacco use and the introduction, manipulation and control of Aboriginal people with tobacco during colonisation represents an important opportunity to counter the narrative of smoking as a personal choice or act of resistance, and to discuss the intergenerational transfer of smoking.
4. *Activities should be provided to address the broader underlying drivers of smoking and other risk behaviours, reducing stress and promoting good mental health and wellbeing.* Although the previous iteration of the Tackling Indigenous Smoking program included a Healthy Lifestyles component with a focus on nutrition and physical activity and some relevant activities such as cooking classes, sport and cultural games [50], programs need to address broader determinants rather than the behaviours of multiple chronic disease risk factors simultaneously. There is a policy opportunity for greater funding flexibility for community organisations to design tobacco control programs that have the capacity to modify determinants of youth smoking, including boredom, disengagement, stress and poor mental health, through structured activities and supports that also build cultural and community connections.
5. *Community-level activities should be reinforced with strong national, mainstream tobacco control measures, including smoke-free legislation and social marketing campaigns, to continue the de-normalisation of smoking.* Social marketing campaigns can play a key role in building knowledge of non-smoking expectations, increasing confidence to reject offers of cigarettes and reducing the appeal and status of cigarettes [51,52]. These campaigns are important for both reinforcing the attitudes and intentions of young Aboriginal non-smokers and for continuing to drive down broader population prevalence. Sustained national comprehensive tobacco control can ensure there is no erosion of the effect of community-level programs.

### 4.5. Strengths and Limitations

There are some limitations to this study. This study includes Aboriginal participants from only two sites in regional and urban NSW, and we acknowledge that given the diversity and heterogeneity of Aboriginal and Torres Strait Islander peoples the findings may not reflect the experiences and views of people from other communities, particularly those in remote areas. There is possible sampling bias, as participants were those engaged in a community-controlled study and available during business hours (or whose parents gave them permission to attend during school time). This may have exaggerated the generational differences; however, in some cases, we were able to compare accounts from the same schools and same communities over time, so we are confident in our findings. Finally, all coding and initial analysis was completed by one researcher (CH); however, MC, who facilitated all sessions (with CH), reviewed the transcripts, codes and summaries and confirmed that these reflected the yarns.

This study has a number of strengths. Young community members’ engagement in the interpretation of the survey findings has not only validated the analysis, but has also validated international evidence of influences on smoking uptake. By privileging the voices of young Aboriginal people, we have been able to identify key opportunities to design effective smoking prevention programs that will enhance adolescent health and wellbeing. A key strength of this research is the predominantly Aboriginal research team who have developed the major themes and recommendations in a collaborative manner.

## 5. Conclusions

Through interactive engagement with young Aboriginal people, this study has identified four key themes that can be understood as objectives for programs preventing youth smoking: continue to change the tobacco environment, promote good health and wellbeing more broadly, build on community and cultural strengths, and support the empowerment and engagement of young people. These should be embedded into community-level programs and led by young people themselves. However, large-scale comprehensive tobacco control measures are needed to ensure that the ongoing de-normalisation of smoking is sustained in the broader environment. Programs need to reach people from very early adolescence and into young adulthood to continue to reinforce non-smoking intentions across the key initiation periods of attending early high school, starting to drink and attend parties, turning 18 years old, and starting work.

## Figures and Tables

**Table 1:** Sample characteristics by yarning session.

Interview	Sex	Age Group	Site	Smoking Status	Size
1	Female	18+	1	Non-smoking	1
2	Female	18+	1	Smoking/ex	3
3	Male	12–15	1	Non-smoking/ex	3
4	Male	18+	1	Smoking	1
5	Male	18+	1	Non-smoking	1
6	Mixed	12–15	1	Non-smoking	3
7	Mixed	12–17	2	Non-smoking	3
8	Mixed	12–17	2	Non-smoking	3
9	Mixed	12–17	2	Non-smoking/ex	7
10	Female	12–18+	2	Mixed	2
11	Mixed	12–18+	2	Smoking	3
12	Female	12–17	2	Non-smoking	2
Overall	17 Female, 15 Male	23 < 18 9 18+		7 Smoking 25 Non-smoking (ex, trialled, never)	32

**Table 2:** Summary of all reasons for smoking/non-smoking by key themes.

**1. Drawing Strength from Culture & Community**		
Non-Smoking	Unclear/Both	Smoking
Strong community connection Community belonging Know about culture Connection to culture Strong community		
**2. How the smoking environment shapes attitudes and intentions**		
Non-Smoking	Unclear/Both	Smoking
Smoke-free home * Cigarette cost * Smoking rules * Cigarette packaging * Anti-smoking ads * Role models *+Legislation* *+Smoke-free cars* *+Generational change* Knowledge of smoking harms *		Family smoke * +Bonding *+Pro-smoking attitudes* Friends smoke * +Try to fit in *+Peer pressure* *+Smoking at school* *+Few consequences at school* *+Smoking teachers* Access to cigarettes * See smokers around * +Smoking role models Look good * Smoking in TV/movies
**3. Non-smoking as a sign of good physical, social and emotional wellbeing**		
Non-Smoking	Unclear/Both	Smoking
Fitness * Sport * Good mental health *+Pregnancy* *+Quality of social relationships*	Live with/raised by mum * Sick family * Friendships * Family relationships * Sex/gender *	Depression and anxiety * Stress * +Anger management *+More smoking while excluded* Moved house a lot Alcohol * Yarndi * +other drugs Age * +Acting older *+Going out, going to parties* Being sexually active Addiction * +Habit & routine *+Family violence* *+Males*
**4. The importance of individual empowerment and engagement in being smoke-free**		
Non-Smoking	Unclear/Both	Smoking
Want good marks * Want to go to school * +Future focus *+Self-efficacy to g no* *+Confident advocacy against smoking*		Bullying Racism Curious * Boredom * +Personal choice *+Rejection of harms* *+Short-term focus* *+Fatalistic view* *+Low parental monitoring* Go to court Youth detention Questioned by police Suspension/expulsion Going to school *

Note: * indicates that the card topic was first raised spontaneously, *+ italicised topic indicates additional item raised,* all other items indicate prompted topics not raised spontaneously.

## Data Availability

No further data are available to ensure participant confidentiality.

## Supplementary Materials

The following supporting information can be downloaded at: https://www.mdpi.com/article/10.3390/ijerph20054587/s1, File S1: Discussion Guide Original and Modified/Adapted; File S2: Card Sorting Activity Topics; File S3: Fieldwork Photos; File S4: NVivo Coding Structure; File S5: Extended Participant Quotes; File S6: Large scale Figure 1.
