# Supplementary material for: Don’t Follow the Smoke—Listening to the Tobacco Experiences and Attitudes of Urban Aboriginal Adolescents in the Study of Environment on Aboriginal Resilience and Child Health (SEARCH)
Source: Int J Environ Res Public Health. 2023 Mar 4;20(5):4587. doi: 10.3390/ijerph20054587 (PMC10002023; doi:10.3390/ijerph20054587)
Supplement: Supplementary file 1 [file ijerph-20-04587-s001.zip › S1 Discussion Guide Original and Modified.pdf]

SUPPLEMENTARY FILE 1.1 – DISCUSSION GUIDE ORIGINAL

- Welcome + reminder of ethics
- Introductions

- What does being "healthy" mean to you? What does it look like?
- What is the opposite?
- What things help or don't help being healthy?

- Where does smoking fit in that mix?
- What have you seen or heard about smoking?
- How do you think most people your age feel about smoking?
- Would anyone like to share any experiences – your own or friends or family?
- What things do you think might mean someone takes up smoking?
- What things do you think might prompt them to quit?
- What about never taking up smoking at all?

- Card sort activity – things that you think may be important for when or how someone like you might start smoking or not.
  - Pull out the three most important things for young people smoking
  - Pull out the three most important things for staying a non-smoker
  - Tell us a bit about the ones you picked out. Any that were not important at all?

- Here were some things that looked like they might be important for being a non-smoker in the SEARCH survey results – do you agree?
  - Good mental health
  - Good family relationships (no arguments, live with Mum or Dad)
  - Haven't moved around a lot
  - Non-drinkers
  - Not sexually active
  - Not been in trouble with school (expelled/suspended) – or other family
  - Not questioned by police, or had to go to court, or friends in juvenile detention/prison.
  - No yarndi use
  - School engagement and positive attitudes about going and good marks
  - Parents don't smoke
  - Strong sense of community where they live
  - More money at home
- Any missing?
- Any that would be more or less important as you get older?

- Thinking about everything we've discussed what's one thing we could do more of to help young people like you grow up as non-smokers?

- Thanks + give out vouchers

SUPPLEMENTARY FILE S1.2 – FINAL DISCUSSION GUIDE MODIFIED/ADAPTED WITH SITE 2

- Welcome + reminder of ethics
- Introductions

- What does being “healthy” mean to you? What does it look like?
- What is the opposite?
- What things help or don’t help being healthy?

- Where does smoking fit in that mix?
- What have you seen or heard about smoking?
- How do you think most people your age feel about smoking?
- Would anyone like to share any experiences – your own or friends or family?
- What things do you think might mean someone takes up smoking?
- What things do you think might prompt them to quit?
- What about never taking up smoking at all?

- Card sort activity – things that you think may be important for when or how someone like you might start smoking or not.
  - Organise into non-smoking, smoking, unsure/could go both ways
  - Pull out one or more you feel are most important for young people smoking
  - Pull out one or more you feel are most important for young people non-smoking
  - Tell us a bit about the ones you picked out. Any that were not important at all or unsure about or could be both?

- Thinking about everything we’ve discussed what could we could do more of to help young people like you grow up as non-smokers and be healthy in general?
  - What would a program look like or involve?
  - Who would it target? Age/smoking status?
  - How often/long would it run?
  - What sort of activities would you want?

- Thanks + give out vouchers
