# Supplementary material for: Don’t Follow the Smoke—Listening to the Tobacco Experiences and Attitudes of Urban Aboriginal Adolescents in the Study of Environment on Aboriginal Resilience and Child Health (SEARCH)
Source: Int J Environ Res Public Health. 2023 Mar 4;20(5):4587. doi: 10.3390/ijerph20054587 (PMC10002023; doi:10.3390/ijerph20054587)
Supplement: Supplementary file 1 [file ijerph-20-04587-s001.zip › S2 Card Sorting Activity.pdf]

SUPPLEMENTARY FILE S2 – CARD SORT

Age	Gender	Bored
Stress	Fitness	Sport
Live with Mum	Good mental health	Depression/anxiety<br>Other mental health challenges
Bullying	Racism	Connected to Aboriginal culture
Know about Aboriginal culture/history	Cigarette cost	Cigarette packaging
Anti-smoking ads	Smoking on TV/movies	Alcohol

Yarndi	Family smokes	Friends smoke
Role models	Smoke-free home	Sexually active
Going to school	Want good marks	Want to go to school
Suspended expelled	Moved house a lot	Strong community
Strong Connection to my Community	Feel I belong to my community	Sick family
Police questioning	Go to court	Youth detention

Addicted	Smoking harms	Curious
Look good	Family relationships	Friendships
Rules about smoking	See smokers around	Access cigarettes
