# Supplementary material for: Don’t Follow the Smoke—Listening to the Tobacco Experiences and Attitudes of Urban Aboriginal Adolescents in the Study of Environment on Aboriginal Resilience and Child Health (SEARCH)
Source: Int J Environ Res Public Health. 2023 Mar 4;20(5):4587. doi: 10.3390/ijerph20054587 (PMC10002023; doi:10.3390/ijerph20054587)

SUPPLEMENTARY FILE S3 – FIELDWORK PHOTOS: Card sorting activity

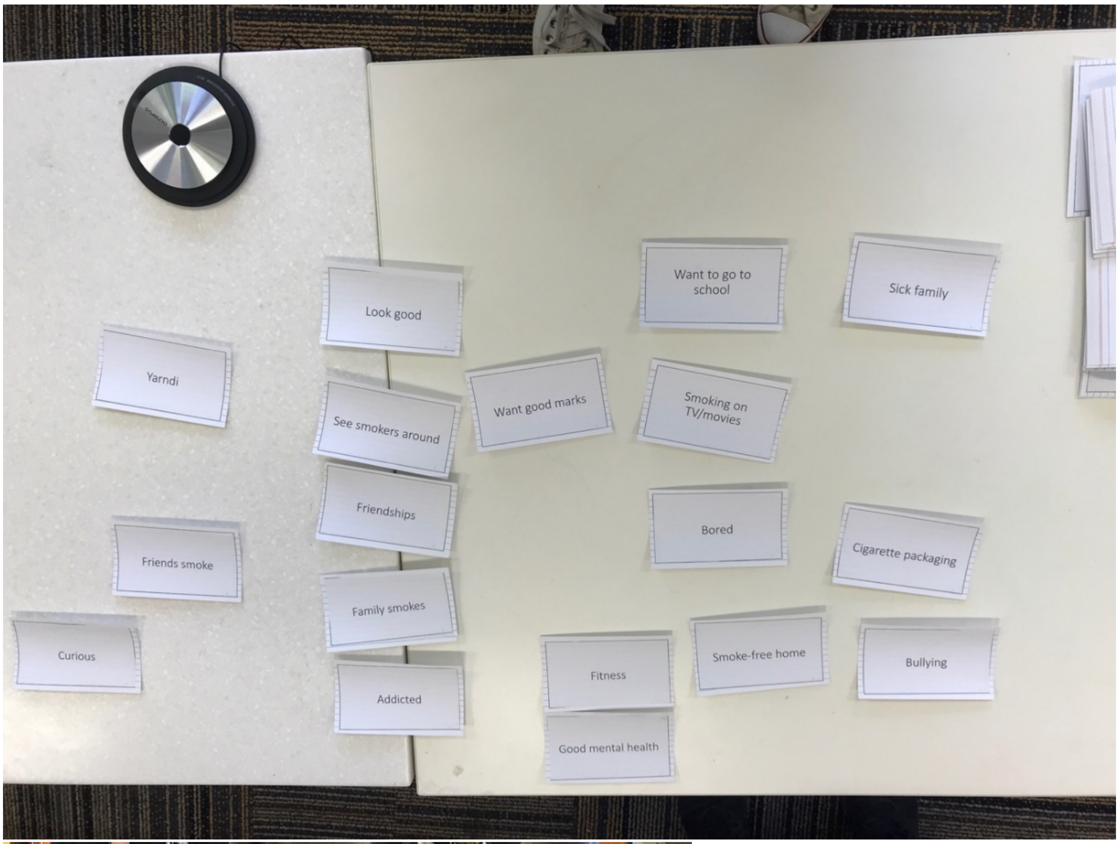

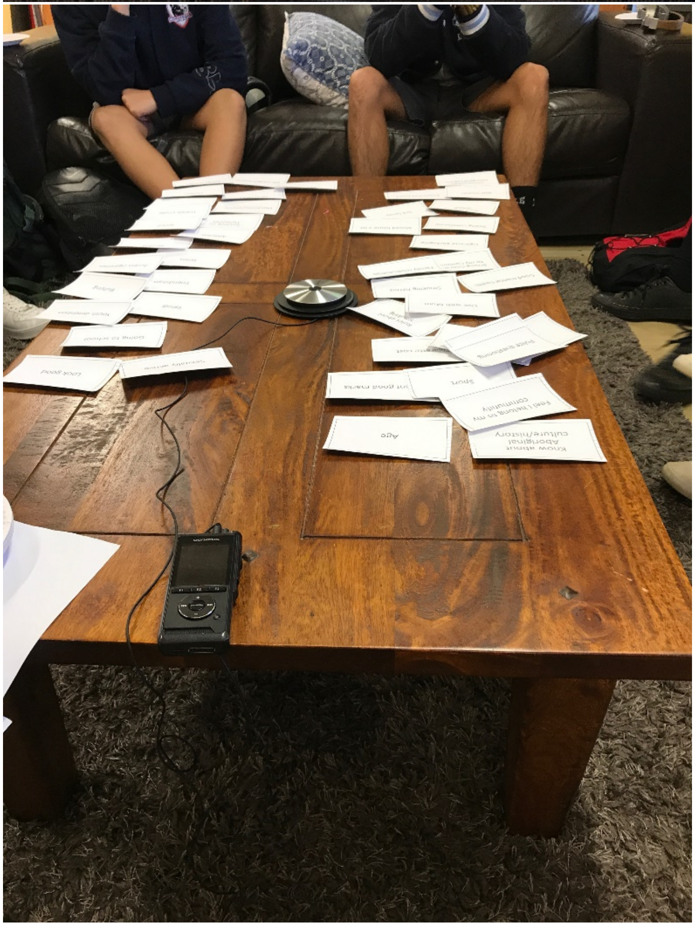

Supplement: Supplementary file 1 [file ijerph-20-04587-s001.zip › S3 Fieldwork Photos.pdf]
