# Supplementary material for: Don’t Follow the Smoke—Listening to the Tobacco Experiences and Attitudes of Urban Aboriginal Adolescents in the Study of Environment on Aboriginal Resilience and Child Health (SEARCH)
Source: Int J Environ Res Public Health. 2023 Mar 4;20(5):4587. doi: 10.3390/ijerph20054587 (PMC10002023; doi:10.3390/ijerph20054587)
Supplement: Supplementary file 1 [file ijerph-20-04587-s001.zip › S4 NVivo Coding Structure.pdf]

SUPPLEMENTARY FILE S4 – CODING STRUCTURE

1. Health in general
   - What is healthy vs unhealthy
     - Healthy
       - Balance
       - Education
       - Employment
       - Good mental health (SEWB)
       - Hygiene and environment
       - Lifestyle general
       - Long life
       - No substance use
         - No drug use
         - Non-drinker
         - Non-smoking
       - Nutrition and Physical Activity
         - Diet
           - Fruit
           - Non-specific healthy food
           - Non-sugary drinks
           - Portion sizes and calorific intake
           - Vegetables
           - Wraps (eg subway)
         - Healthy body weight
         - Physical activity
       - Social connections
     - Unhealthy
       - Diseases and health consequences
         - Cancer
         - Diabetes
       - Kids sick
       - Lack of parental supervision
       - Not able to do what you want in life
       - Not accessing culture
       - Poor mental health
         - Negative mindset
       - Poor nutrition or physical activity
         - Diet
           - Confectionary
           - Eat out or takeaway, fatty foods
           - Sugary drinks (juice, soft drinks)
           - Unhealthy foods general
         - Lack of physical activity
         - Obesity
       - Substance use
         - Alcohol
         - Drug use
         - Smoking
       - Unhealthy general
         - Don't care about looking after themselves
       - Violence (incl DV)
   - What makes it easier or harder to be healthy
     - Barriers
       - Addicted (food, substance use)
       - AMS appointment times
       - Food - convenient, fast, unhealthy, cheap food options
       - Kids
       - Lack of knowledge
         - Sources
           - Friends and colleagues
           - Online
       - Personal choice
       - Poor role models or deviant relationships
       - Safety
       - Stuck at home
       - Time constraints
     - Enablers
       - Access to healthy food
       - AMS programs etc
       - Community Safety
       - Gyms
       - Healthy role models
       - Time management

  - Distance to service
  - Routine
  - Wait times
- Transport

2. Smoking - general
   - Attitudes and beliefs
     - Confidence to verbalise attitudes (share views and influence others)
       - Saying No for self
       - Telling others why they shouldn't smoke or to move away
     - Future aspirations (not smoking specific)
     - Knowledge
       - Awareness of information sources or support services
         - Family
         - Popular culture
         - Posters or social marketing (incl packaging)
         - Quit supports and other support services
           - AMS or doctors
           - Apps
           - Other support services
           - Quitline
         - School education & supports
         - Sport
         - Unsure
       - Harm to others
         - Burden on family
         - SHS exposure
       - Harms to self
         - Financial impacts
           - Cigarette cost
         - Loss of future opportunities
         - Loss of time with family
         - Perception of differential harm and intensity
         - Physical health impacts
           - Cancer
           - Cosmetic (teeth)
           - Damage lungs and breathing problems
           - Death
           - Impacts fitness
           - Unhealthy general
       - Historical smoking
     - Negative attitudes
       - Attitudes to smoking of others
         - Disgusted
         - Hurt and upset
         - Ignored
         - Image
           - Shame or embarrassing
           - Uncool
         - Silly
         - Stupid
         - Too young
         - Waste of money
         - Wrong
       - Bad habit
       - Gross
         - Coughing or choking
         - Smell
         - Teeth
         - Yuck - feel yuck or sick
       - Impact friendships
       - Not for me
       - Not worth it (cost, smoking restrictions - getting too hard)
       - Regrets about own smoking
     - Perceived reasons for smoking or not
       - Access to cigarettes
       - Addicted
       - Age
         - Adulthood aspirations
       - Alcohol and other drug use
         - Cannabis use
         - Drinking
         - Drugs general
       - Boredom
       - Curious or experimentation

            - Domestic Violence
            - Don't know
            - Family factors
                - Family smoking
                    - Bonding
                - Parents don't know
            - Fatalistic view (we're going to die anyway)
            - Friends smoke (incl colleagues)
                - Peer pressure
                - Self image (think they're cool, try to fit in, rebellious)
            - Gender differences
                - Males
            - Generational change
            - Habit or routine
            - Knowledge or rejection of harms
            - Legislation and rules around smoking
            - Mental health
                - Depression
                - Stress & anger management
                    - Other (music, fishing)
                    - Smoking
                    - Support services
            - Personal choice
            - School factors
                - Few consequences at school
                - Smoking teachers
                - Wagging class
            - Short term vs long term focus
            - Social influences general
                - Society
            - Social smoking (e.g. parties, work)
        - Smoking relative to other health outcomes
    - Experiences and intentions
        - Non-smoking personal experience or future intentions
        - Pro and neg exposure or attitudes from others
            - Family
                - Non-smoking
                - Smoking
            - Friends or peers (incl colleagues)
                - Non-smoking
                - Smoking
                    - Age groups
            - SHS and smoke-free rules or legislation
                - Avoid personal exposure
                - Distance from restaurants, bus stops
                - Exposure to kids
                - Parties
                - Smoke-free homes or smoking at home
                - Smoke-free pubs clubs
                - Smoking at school
                - Smoking in cars or smokefree cars
                - Visibility in community
                - Workplace
            - Vaping
        - Smoking personal experiences intentions
            - Access
            - Co-existing health problems
            - Current Smoker
            - Initiation
                - Age smoking commences
            - Intensity and frequency
            - Other behaviours
            - Pregnancy
            - Quitting or not continuing to smoke
                - Relapse
            - School
3. Smoking - specific prompted card sort responses
    - Access to cigarettes
    - Addicted
    - Age
    - Alcohol
    - Anti-smoking ads
    - Bored
    - Bullying

- Cigarette cost
- Cigarette packaging
- Connected to Aboriginal culture
- Curious
- Depression, anxiety, other mental health
- Family relationships
- Family smokes
- Feel I belong to my community
- Fitness
- Friends smoke
- Friendships
- Gender
- Go to court
- Going to school
- Good mental health
- Know about Aboriginal culture and history
- Live with Mum
- Look good
- Moved house a lot
- Police questioning
- Racism
- Role models
- Rules about smoking
- See smokers around
- Sexually active
- Sick family
- Smoke-free home
- Smoking harms
- Smoking on TV or movies
- Sport
- Stress
- Strong community
- Strong connection to my Community
- Suspended or expelled
- Want good marks
- Want to go to school
- Yarndi
- Youth detention

4. (dropped later groups) Smoking - specific prompted survey checking
5. Program ideas
    - Features
        - Compulsory attendance
        - Diversional activities
            - Computer access
            - Cultural
                - Art class and street art murals
                - Camp
                - Cooking class and traditional food recipes
                - Cultural awareness and knowledge general
                    - Aboriginal pictures on posters
                - Cultural dancing
                - Cultural games
                - Cultural music (learn to make and play didge)
                - Jewellery making
                - Women's group
                - Youth group
            - Game night
            - Movie night
            - Music festival, recording or disco
            - Non-team physical activity (yoga, swimming, dancing)
            - Sport
        - Food
        - Incentive (Koori designed merch for participants)
        - Interactive learning
            - Fun
            - Hands-on
        - Role Models
            - Personal stories
        - Support group
        - Writing
    - Objectives
        - Awareness (prevention general)
            - Information and education sessions
        - Quit attempts

- Skills building and reducing other behaviours
  - Future or general whole of life focus
  - Healthy nutrition and lifestyle
  - Manage peer pressure
  - Mental health & stress management
  - Other substance use
- Settings
  - Advertising
    - Media
    - Pamphlets
    - Posters
  - Community
    - Event
  - Health professionals
  - School
  - Sports clubs
- Target audience
  - Age
  - Behaviours
  - Gender
- Timing
  - Duration
  - Frequency

6. Summary
   - Summary non-smoking influences
   - Summary smoking influences
   - Summary undecided or neutral or both
7. Key quotes
