# Supplementary material for: Don’t Follow the Smoke—Listening to the Tobacco Experiences and Attitudes of Urban Aboriginal Adolescents in the Study of Environment on Aboriginal Resilience and Child Health (SEARCH)
Source: Int J Environ Res Public Health. 2023 Mar 4;20(5):4587. doi: 10.3390/ijerph20054587 (PMC10002023; doi:10.3390/ijerph20054587)
Supplement: Supplementary file 1 [file ijerph-20-04587-s001.zip › S5 Extended Participant Quotes.pdf]

SUPPLEMENTARY FILE S5 – EXTENDED PARTICIPANT QUOTES

Notation guide: Participant quotes are presented unitalicized with the participant indicated by [Interview Number (1-12) . Gender (M or F) and a number indicating they were the nth male or female participant in the group] e.g. [5.M1] refers to a quote from interview number 5, with a male participant who was the first male to contribute in that interview. *Interviewer questions are italicised and individual interviewers indicated by I1 and I2. Additional contributions/questions from other SEARCH Aboriginal Research Officers / Aboriginal Health Workers are indicated by RO, AHW.*	
General concepts of health and wellbeing	Um obviously like smoking and that, drugs and stuff. I don't really like doing that stuff. Like, it's a turn off. When you see other people like out in the community, like sit there like doing it and that, it's a turn off to me. [5.M1]
Personal exposure and experiences of smoking/non-smoking	Fifteen. But only one. Didn't like it. *[I1] And why was that? So, you didn't like it? How'd you feel after you first, after your first try?* Like it. I don't know, like I felt different. It was just kind of like a peer pressure thing cos everyone around me was doing it.<br>So I ended up trying it. It affected me badly for one day. And a couple of days after. *[I1] Where was that, at school?* Yeah. *[I2] What do you mean by affected you badly, did you feel sick?* Yeah. Like I felt like a bit light headed and everything. Got me really bad cos I never did it, but like everyone around me smoked, cos I didn't. It got me. Short breath. With running, definitely. *[I1] And have you had a cigarette after that?* No.<br>*[I1] That's great, that's good. So, so just going through that stage of feeling very sick and very ill turned you off? Turned you off, turned you away from smoking.*<br>Yeah, I try to convince my parents and siblings to like to stop it, from what happened but they, they didn't really get what I had. So. [12.F1]
	[I1] Anything else you'd like to share? Any other stories? Any other thoughts on cigarettes? When you're going to give up?<br>One day. [11.F2]<br>I go on and off. I think I only smoke due to my peace, that's like my peace. You know. My little getaway from my little one and partner and yeah. And I think I smoke.<br>I really do, I just can't find myself to quit right now. When I become more stable, when I get my own house that I know I'm not going to get kicked out of. And when I get the kids settled, and like I can start focusing on my health again. I really want to quit, I just can't right now. I think once I get more stable, and it'd be easier for me to quit. [11.F1]<br>*[I1] It's never easy. I'm a smoker, and yeah, it's the worst thing I started ever.*<br>Yeah, I kind of regret starting but now that I've started I can't stop. [11.F2]<br>*[I1] Yeah, I was eighteen, so a lot of my influences were from my cousins, but something I deeply regret. A lot of family have passed away, that I've lost through cigarettes, but yeah, the addiction. Yeah, I've tried four times to give up. Um, I'm not a big smoker these days, so just do what they call routine, or relaxing, smokes. So one in the morning and one at night, but yeah. I smoke a lot less than what I used to. Yeah. But I encourage youse to try and make some changes, cos obviously you know the consequences as you get older. And especially you, bud, at your age. Try to, try to start saying no to it, yeah? Cos yeah. There's a lot of things I struggle with now, and I'm only, you know, I'm only forty years old, and I'm struggling because of cigarettes. Twenty two years of my life, and I always kept saying I will stop one day, I'll stop one day, but yeah the older I get the worse it is for me. Like, I struggle to walk two hundred metres up the road, and play with my kids. So, yeah, a lot of lifestyle changes need to happen for me.*<br>Yeah, I struggle walking down the mail box. [11.F2]<br>*[I1] At a young age, you just keep going, and "one day I'll stop".*<br>So I've got half my lungs, half my lungs filled with asthma and the other half's filled with smoke. [11.F2]<br>*[I1] Yeah, so it's starting to scare the shit out of me. Yeah. So um, yeah, two a day, at, at, when I look back three years ago, two a day for me is really, really, is really really good. But I know there's still too much. So I would encourage the three of youse, even just as a family, to sit down and start talking about bout when, when do we stop.*
**Theme 1. Drawing strength from culture and community**	
1.1 Community, cultural connections and role models	I reckon, just like if you're someone that's deeply connected with your culture, it gives you like um, I don't know, just like a sense of belonging, and it just makes you not want to smoke because you're more interested in, in your culture. But like, so could be either studying, it could be working with the community. It could be um, studying your culture. Like yeah, and just, I feel that being connected to your culture, it does um make you want to smoke less, just because of wanting to fit in with the, the rest of your community and stuff like that. [11.F2]<br>*So, just having that just correct me if I'm, tell me if I'm wrong, but is it sort of being engaged, and being occupied during the day sort of just giving you like [I1]*<br>Yeah, with other blackfellas, talking about your Aboriginal heritage and your culture and stuff. Yeah. [11.F2]
	Cos I think the um, you have to, to have a strong connection to my community, um, I have to like be a good role model for um, cos I work with families. So I have to be a good role model for them families. Um, I also work with children, so it's good to be a role model for the children… Yeah, and I think if you do have them good role models, that's more likely to change people's opinions and, on certain, on certain issues. [10.F1]
	Role models. Just don't be a follower, be a leader. And then strong community, ah, connection to my community. So, keep everyone in the community strong, try and quit smoking. Be healthy. Yeah, live longer. [4.M1]

Theme 2. How the smoking environment shapes attitudes and intentions	
2.1 Role models	*[I1] [MALE TEACHER] would be a really good mentor at school?*<br>Yeah. Yeah he is. [8.F2]<br>He is to all the kids, like there's a couple of Aboriginal kids that don't smoke any more there now, cos of him. [8.F1]
	Like role model, like it's in there, but I feel like it can kind of be wrong, cos if your role model smokes, you're going to kind of want to be like him. [7.F1]
2.2 Friends who smoke and smoking at school	I think it's also like peer pressure. I mean they see someone, so they say you want a draw now? And it's no you've got to do it. Yeah, you just kind of want to fit in kind of thing. Follow the leader. [2.F2]<br>*[I1] So you mention it becomes an struggle if you say no.*<br>Yeah, it's going through your mind "what are they going to think of me if I say no". [2.F2]
	Yeah, I've tried cigarettes but I don't smoke, occasionally. Like when I'm around parties and all that, it's kind of a social thing where you feel pressured to smoke, in front of everyone. And so obviously, I just usually do it at parties I think. [10.F1]<br>*[I1] So, at parties, majority of your friends?*<br>Oh, yeah. They're all smoking. Everyone's smoking. [10.F1]
	[I1] So knowing that Nan had cancer, what made, what made you want to smoke?<br>Mostly just people around me. I don't do it all the time, you know, just mostly when I'm around people. [10.F1]<br>*[I1] Ok. So just influences is the reason why?*<br>Influences. Yeah. [10.F1]<br>*[I2] So you know how you said you feel the pressure to smoke, does anyone actually say anything to you, or is just kind of like, it just feels like everyone else is doing it, and like no one's actually saying "come on, have a cigarette", it's just kind of the general vibe?*<br>Um, no, sometimes, people, I'll have a friend sitting there and she'll you know, be sick of people telling her "oh, just have a smoke" never smoked before "have a smoke". And I'm like "no, don't". I'm a good friend I won't let her smoke. But she kind of smoked that day. Um, yeah, they, they pressure you sometimes. [10.F1]<br>*[I2] Really?*<br>Yeah. [10.F1]<br>*[I2] Why? If someone says that they don't want to?*<br>Oh, just stupid kids. "Oh, nobody's going to smoke with us", and silly. It is. [10.F1]<br>*[I2] Did you ever get the, the pressure that [F1]'s talking about?*<br>Not so the "here have a smoke", but more so um, just letting you in sort of like a little circle, and everyone else is doing it, and you just sort of feel left out. [10.F2]<br>*[I2] Yeah, kind of like a way to be part of the group.*<br>Yeah. [10.F2]
	[RO] Do you think that your main reason for starting was just because of your friends? Yep?<br>And now I've got no friends I've only got me and my two kids [2.F1]<br>*[RO] Kids. Yep.*<br>It's nothing to be proud about now. [2.F1]
	But when I became an adult and started working and stuff, and started hanging round people that did smoke, I did end up picking it up. So I shouldn't even have started. I've only been smoking for about four or five years, but since I have been smoking, I've gotten pretty bad at it. [11.F2]<br>*[I1] Um, how'd you feel after your first cigarette?*<br>I started choking a lot. Like, I couldn't really handle it, that was then, and then I started a lot. But um, yeah. I started smoking probably only socially. So I'd go and just have a yarn with the girls, I'd have a smoke. And then soon it started me going out by myself to smoke. And then I just started going again and again, and I just never stopped. [11.F2]
	Everyone just thinks like they're wannabees. But then they think to themselves "oh, we're top shit" and all that. [7.F1]<br>*[I1] But across the board, no one likes it? Like within your age group?*<br>Yeah, no one likes it. [7.F1]<br>I think it's embarrassing. If I was looking at it and saw someone, I'd be embarrassed if I was them. [7.M1]
	[I2] What's, why would you be trying to look cool by smoking? What would be the purpose of that?<br>I don't know, I just feel like they like want to be older than they are. You know. [7.F1]<br>*[I1] Yeah?*<br>They think that they're like seventeen, but they're like twelve, fifteen [7.F1]
	I just think most people do it to look cool. Like I don't think they really think through when they smoke, what it's actually doing to your body. [9.F1]<br>Trying to show off. [9.M1]
	But most of them, most of the year sevens hang out with year twelves, so that they can buy smokes. Year twelves can buy smokes from there. So that's where they get the smokes from. [8.F1]<br>The teacher smokes, so [8.F2]<br>It can really put an influence on the kids [8.F1]<br>But the teachers go outside the school gate and, or some of them, or some of them waits until after school. [8.M1]<br>*[I2] Yeah. Do you reckon the teachers feel like they can't really say much about it if they're smokers themselves?*<br>Yes. [8.F2]

	Harsh. Nah! [8.M1]<br>Yeah, plus some of the teachers are scared of us students because most of them hit um, the teachers. [8.F1]<br>*[I1] Students do?*<br>Yeah. [8.F2]<br>*[I2] Sounds hectic.*<br>It is. This school is one of the worst. [8.F1]<br>The most students that smoke. [8.M1]<br>Because they can, I guess. [8.F1]<br>Now it's just a bad school, bad environment to be like taught in. [8.F2]
	[I1] So, um, how does it make you feel though, when, when you sort of know the effects, and having that experience of having a cigarette, how does it make you feel knowing that they don't want to listen?<br>Oh, makes me a bit upset. You could like kind of take all that advice from someone and then try and give it to someone else, to try and prevent them from…stopping what they're doing, or their habits, negative habits such as smoking, or anything. Negative habits such as drugs, alcohol. But like I said, you can't really force them to stop, as much as you try, they're not going to listen to you regardless. So it does hurt a bit. [12.F1]<br>*[I2] Those conversations are hard, has it affected your friendships at all when you've had those conversations?*<br>It has to a few, but you gotta learn to move on. You get over, you win some you lose some. Just gotta flow from that thing. [12.F1]
2.3 Smoking among family members	My mum's a big smoker too. She started smoking when she was about twelve. Same with my brother. His father, he started smoking when he was about twelve. And my sister, she started smoking at a young age too. But because I had health problems, I didn't start smoking till later in life. [11.F2]
	My mum is a smoker, and her mother is a smoker, and so was her mother. So um, I don't know, it's just like [11.F2]<br>*[I1] So it's a generational factor?*<br>I guess, yeah. Um, [11.F2]<br>*[I1] Mum's always been a smoker?*<br>Yeah, Mum's always been a smoker, since a young age, and so was her mother, well now me and my brother and sisters smoke, and my brother's kids smoke. And it's just yeah. I don't know. It just, like I don't know I don't know why I put it in that pile. [11.F2]
	But my son, he's only two, and his nan like came down from [Town2] and she just, she was staying at my house and she kept going out the back smoking. And after she left, he'd get up and be like "mum I want a moke". That's what he said. [1.F1]<br>*[I1] Hmm,*<br>*[I2] Ohhh*<br>That's what he said. And I was like "what?" And then he's like "I want a moke". But see, cos he noticed her smoking. Cos like nobody else in the house smokes, but seeing her kept, keep going outside, and then he probably thought he'd be able to go outside if he smoked. Like that's, it shocked me when he said that. [1.F1]
	[I1] What were your thoughts though when you seen your mum and that smoking?<br>Oh, I thought well "like, why do you smoke? Don't do that, I don't want you to die" or something, you know what I mean? [4.M1]<br>*[I1] Oh, ok.*<br>And then now I'm doing it. Only when I drink though but still, I don't reckon I should do it, cos, it's not a healthy thing. [4.M1]
	[I1] How does that make you feel, like having a night out with cigarettes?<br>I feel normal. It's gross. They are, they're gross. Um, most of my family smokes, not any more. My Nan used to smoke, she got cancer from it. Um, I used to hide her smokes from her. She used to get really angry. It was really funny. You know, it was a good thing. I used to, cos she had cancer, she wasn't supposed to smoke. So. Yeah. [10.F1]<br>*[I1] So knowing that Nan had cancer, what made, what made you want to smoke?*<br>Mostly just people around me. I don't do it all the time, you know, just mostly when I'm around people. [10.F1]
	Oh my parents told me. [6.F1]<br>*[I1] So, what do they say?*<br>Oh, they go, "if you ever get home, or if I get a phone call to say you were smoking, you're gonna get flogged." I'm like ok. [6.F1]
2.4 Smoke-free homes and second-hand smoke exposure	It's very important. Teach your kids not to smoke. Give them the healthy lifestyle. [2.F1]<br>*[RO] I think too, like the passive smoking. Like having a smoke free home, you don't, you're not exposing your kids to it as much as what you would if you were to be smoking inside. Cos they don't really get a say. Yeah, you choose to smoke but your kids don't choose to inhale it.*
	[I1] What's it like for you two at home?<br>Um, our dad smokes. Yeah. Most of his family does. Like um, grandma, poppy, and all that. Um, yeah. [9.F1]<br>*[I1] And do they smoke inside the home?*<br>Nah, outside. [9.F1]<br>*[I1] When people come to visit your house, do they generally smoke outside as well?*<br>Yeah. [9.F1]

	[I1] And do they ask to smoke?<br>Oh, yeah, they ask if they can have one and go outside. [9.F1]
	Upset to be honest. Like, like if they're smoking round me, like you said before, I didn't ask for that to come onto me, no. Yeah. I don't know, write up a school program or something. [9.F2]
2.5 Knowledge of harms and impacts from smoking	*[I1] Hey. And what it all comes down to is I suppose this is sort of a message from myself, is um, if we don't smoke cigarettes at all a lot of these factors go away. A lot of them go away.*<br>So if youse don't smoke, you don't smoke. You're free of it. [8.FF2]<br>You save a lot more money. [8.F1]<br>Not even that. If you don't smoke, you're saving money, and you're saving yourself from like that mental, that unhealthy state of life. Yeah. [8.F2]
	I think that's the main reason I don't do it. Cos you know, you see ads and that on TV. Um, yes. My biggest turnoff. Lung cancer and that's a big thing. Don't want to do it. Cos you put your family through a lot and that. [5.M1]
	[I1] Is that sort of the only awareness you see? Do you see anything with TVs and that? Oh, ads and that. Yeah, I see ads and that, like quit smoking, call smoke line, or help line or whatever it is. I don't know what it is. I seen it on ads. [4.M1]<br>*[AHW] Do you see much though?*<br>No I hardly see stuff about smoking to be honest. Only thing I see is all the stuff from the packets of people like dying and yeah. That stuff. That's the main thing I see on the smokes. [4.M1]<br>*[I1] What do you think when you see those?*<br>It's horrible. [4.M1]<br>*[I1] Makes you not want to smoke.*<br>Yeah, it does. It does. I don't want to end up like that. Know what I mean? I don't want anyone to end up like that. [4.M1]<br>*[AHW] Foot cut off and stuff.*<br>*[RO] Blood on the tongue.*<br>Yeah, stuff like that. Lad was like a fit fella looking healthy, and then just skin and bones. [4.M1]<br>*[I2] That's 'Brian'*<br>Yeah, Brian that's the one I'm talking about. You knew who I was talking about, eh? [4.M1]<br>*[AHW] The one with the bald head? Yeah I think I've seen him.*
	[I2] Um, hey [M1], with your uncles, do you reckon if they knew what you knew, they would have been encouraging you? Or was it more like they just wanted to share and bond?<br>They wanted to share. [11.M1]<br>*[I2] Yeah. So they probably still had a bit of knowledge, but it's more about bringing you into the circle?*<br>Yeah. [11.M1]<br>*[I2] Didn't want to make you feel left out?*<br>Yeah. [11.M1]<br>*[I1] Interesting. Interesting. And yeah, and, and none of that's your fault, bud. That happens everywhere. Like I was, that happened to me as well when I was very young. My uncle did that to me, but when I went straight inside and told my Nan, my Nan chased me with the broom. Um, but that was my first introduction to a smoke, at twelve years old. He was "come here and try this". And I coughed up and coughed up. I spewed everywhere, and my Nan said "what are you doing spewing out the back", and that's when I just spat it out. I can still see her running past the chickens going….. But yeah, so it does happen.*
2.6 Historical knowledge and awareness of policy or legislation change	*[I1] Yeah? And you wish you had ever, do you wish that you never ever took it up?*<br>Yeah. [11.M1]<br>*[I1] So if they were in place when you first started smoking, do you think you were more likely to smoke, or less likely to smoke?*<br>I'd like to think less likely. But yeah. Cos of all the, like all the um, things in place now about being able to smoke here, there and everywhere, I probably would of, back then. You had more freedom back then. And the smokes were cheaper back then. They were cheaper and you were allowed to smoke in more places. You know, there weren't that many rules, but now there's so many rules around it, and they're so expensive, and it's just, it's starting to feel like it's not worth it anymore, but it's really hard to quit. [11.F2]
	So, you know how you said smoking was all round you, how did you cope with it? Like what did you do? [9.F2]<br>*[I1] Um, well I don't know. It was hard. Like there was not much you can do. The laws were different back then, so laws, you were allowed to smoke in the house, you were allowed to smoke in the car. You're allowed to smoke in an office. You were allowed to smoke in a café. You were allowed to smoke in a pub.*<br>Did you end up getting pressured a lot? [9.F2]<br>*[I1] No, so I smoke, I smoke. I started smoking when I was eighteen and that was through influence. But um, you know, this is twenty five years ago, so a lot of people, it was just everywhere. So it was very hard, um to say no to it really. Cos it was just, you'd go*<br>Like to parties? [9.F2]<br>*[I1] Yeah, I think it was probably easier at a party. If you go to a party, there was always sectioned off, like there was smokers down near the, you know, the yarndi smokers there, the spirit drinkers there, the beer drinkers there. So at a party it was good, cos you didn't get around it. Um, but yeah, when I turned eighteen it was really hard cos I was where all the adults were, and yeah, it's something I don't, I don't encourage anyone else to do cos it is, this here is what it becomes, is this here. It becomes addictive, yeah. So even though my personal story, there's been four occasions where I've tried to give up, um, these days it's a lot easier because people, a lot of people don't talk about smoking, and the advertising's not around*

	as much. And obviously, like I said, the new laws that have all changed. You know, you can go to smoke free environments now everywhere. So it's very easy to,<br>So, the laws we have now and that, do you, like wish they were there when you were little? [9.F2]<br>*[I1] Yeah, definitely. Yes I do, yeah. That's a good question. You should be a reporter.*<br>*[I2] That was an excellent question.*<br>*[I1] Yeah, yeah it is. But these are things that I sit back actually, and think on, if all these things that are part of your generation now, the laws, and restrictions, if that was in place when I was younger, then yeah. Cos, you know, when I look back on it now, um, majority of my elders have all passed away from cancer, from cigarettes. So, very preventable diseases. Um, if the laws had changed many, many years ago, then a lot of my elders would still be alive today. I know that for sure. And the fact that cigarettes and alcohol was an introduced thing to our people you know, makes it even, um, makes it more upsetting in a way. There was no need for our people to have these influences given to us.*<br>So, when you're um, when you're like, your uncles pass away from smoking, cancer or something, did you look on that and try quitting? Once? [9.F1]<br>*[I1] Yeah, yeah. Definitely. Um, yeah, all my uncles, the majority of my mum's sisters, they all smoke except for one. All my mum's uncles smoke. On my dad's side of the family, there's only two siblings that smoke. But, you go back to the generation before that, all my grandparents and all their siblings, they all smoke, and drink. So slowly it's sort of changing, within my sibling groups, there's only myself and my younger brother that smoke, and the others don't. But yeah, I just, if the laws had changed a lot back two generations ago, I know a lot of my family members would still be alive today. Still be alive today. Cos yeah, as I was saying earlier, with the campaigns back in the eighties and the nineties, were all about cigarettes, go and buy a cigarette. You know, there was a lot of actors, we had one famous actor, James Dean, he was always, he had a photo with a cigarette in his mouth. And James Dean was a real cool famous actor. But it's just during all that, during that sort of era…*
2.7 Vaping	Vaping's stupid. What's the point of that? *[Mimes cloud of smoke]* [6.F1]<br>*[I2] Maybe they think they look like a magician or something.*<br>Yeah, like, they reckon they're mad. They make themselves shame. So shame. Dumbest thing ever created. [6.F1]<br>*[I2] I think it's weird too. They've got the whole kit, and they unpack it.*<br>*[RO] And all the flavours. They have different flavours.*<br>*[I2] Um, do you see many people vaping in [TOWN]? I haven't seen anyone.*<br>Nah. [6.M1]<br>*[RO] No, we never see it really.*<br>*[I2] I saw it at the shop, but*<br>Every now and then but not all the time. I see someone what an idiot, how stupid is that. How stupid is it to just stand there, inhale some smoke and blow it out? "Oh because it tastes nice." It's so stupid. [6.F1]
	[I2] I was interested to know about vaping.<br>Yeah, she's got a point there. Vaping. Lot of that around now. [9.M2]<br>*[I2] Really?*<br>Can vaping cause cancer? [9.F1]<br>*[I2] We don't know yet. It's too new*<br>And Shisha as well. [9.M4]<br>There's a lot of that going around. [9.F2]<br>A lot of people at night go do shisha. [9.M4]<br>People bring that to school too [9.M3]<br>*[I2] They bring the vapes to school?*<br>Yeah. [9.F2]<br>*[I1] It's a very unknown thing.*<br>Especially when it blows up in your face. [9.M1]<br>*[I1] There's a lot of things. For me personally, I wouldn't do it, I wouldn't do it because there's still a lot of research around that area. Um, when it comes to things like these, especially this society, these days, this is just a craze. So unfortunately it's just going to hang around. So people will do their vaping craze and then they'll move on, but then there'll still be people um, come through later life and still do it, unfortunately. But yeah.*<br>*[I2] The ones that are bringing…*<br>*[I1] Well, how does it, how does it make, how does it make youse feel when you see people, what is it, vaping? They call it? How does it make youse feel?*<br>I just laugh, I don't know. [9.M3]<br>*[I1] Yeah?*<br>*[I2] What are the devices that they have? Cos I know there are some that look like little USB sticks, and*<br>Yeah, the JUULS. [9.M3]<br>*[I2] Yeah, is that what they have?*<br>Yeah. Hmm. You can also get them, it looks like a pen. [9.M3]<br>*[I2] Oh, really?*<br>It's called a vape pen. Yeah, they just, they just have the different flavour too eh. [9.M3]<br>There's a strawberry one and a coffee one. You know, them drinks. [9.M5]<br>*[I2] That's how you get around the like, disgusting smell and taste, if they've got like*<br>Yeah, yeah. [9.M3]<br>*[I1] I've seen videos of them, but just yeah. Is it, does it happen a lot here? Vaping?*<br>Yeah, they all try to blow circles. [9.M4]<br>They even do it in class. [9.M3]

	The teacher will be writing on the board and they'll go and then put it in their bag. It's funny. [9.M2]<br>*[I2] What.*<br>*[I1] So, do youse regard vaping as smoking?*<br>Yeah. [9.F2]<br>*[I1] You do?*<br>Yeah. [9.M5]<br>*[I1] Yeah?*<br>*[I2] Do you think that people think that it's, I know you asked the question about whether it was as harmful, do people have any idea about whether it's harmful or not?*<br>Um, people who do vaping think it's less harmful than cigarettes. [9.F2]<br>Yeah, they say it's just water vaping. Just water. [9.M5]<br>Or just like air. [9.F2]<br>No nicotine in them. [9.M5]<br>*[I1] So yeah, that's the scary thing about it. It's just that there's a lot more research that's still being done about all that. Um, yeah.*
Theme 3. Non-smoking as a sign of good physical, social and emotional wellbeing	
3.2 Sport and fitness	And if you're, for sport, if you're in a rep team, or if you want to be big in a particular sport, sporting, ah, smoking [7.M1]<br>Won't help you. [7.M2]<br>Won't help you. It'll just make you go down track, and it'll just be bad for you, in anywhere you want to be. [7.M1]
3.3 Mental health and stress	*[I2] So when someone starts smoking, it's not just about the smoking? You think about them in a different way?*<br>Yeah. [5.M1]<br>*[I2] What other things do you think about, is going on in their lives if they've started smoking?*<br>I could, like I'd worry about what's going on with their family and stuff. Start asking questions and that. Yeah. [5.M1]<br>*[I2] I think that's where some of this is coming in.*<br>Yeah, there's always a reason they're doing it. Starting smoking. There's always a reason. [5.M1]
	Most teens smoke in that school, but the main, the main focus around the school is like depression and that. Smoking's not really, but some teens smoke and drink and do drugs, thinking it's going to fix their life up. That's not going to. [8.F2]<br>*[I2] Do you think there's a, you can see a relationship there between the depression and the smoking?*<br>Yeah, cos I go through bad depression myself. So I understand why they do it. I would never, I would never pick it, but I'll fight depression, but I won't go smoke and try and give my life up. That's just bad. [8.F2]<br>*[I2] What are the other ways that you manage your depression then? That people could learn from you. What are the different things that you do instead of smoking, when you're trying to make yourself feel a bit better, and?*<br>I just forget about the bad and like focus on the good. [8.F2]
	[I2] With the stress, is it, does it help with the stress? Or is it just kind of automatic, like is it a chance to have break, or does it make you feel more relaxed? Like, what is it that helps you with stress?<br>A bit of both. Like I know the kids can't come near me if I'm having a smoke. Cos I go outside and smoke, like "you stay inside, I'm out here smoking, leave me alone". [11.F2]<br>*[I2] Ah ha. Little private bubble.*<br>That's why, five minutes for me to sit down and what I've been told, to have a smoke, you literally physically have to calm down because if you're running, or if you're walking around, you can't smoke as well. Because your lungs are working to help you breathe. So, you've got to sit down, relax so then you can inhale the cigarette. It is forcing you to sit still and to relax, just so you can smoke a cigarette. That's, yeah, so to have a cigarette you need to relax yourself, and yeah. I use that a lot. [11.F2]<br>*[I1] I must admit, I've never seen someone running and smoking at the same time.*<br>That's true. [11.F1]
	[I1] Right. um, anything else on this table in whatever spot they are that you'd like to talk about, and why? Like, the importance of them. Something else that sticks out?<br>I think probably moving house a lot. [11.F2]<br>Oh, that was mine. I was sick of moving. Cos I always moved around a lot. My parents, my parents are strict parents. That's why I used to go hiding and do my smoking on the side Um, they still are strict. So I said once I turn eighteen, I was moving out. And then I did, and I just been, I was hopping house to house. [11.F1]<br>*[I2] Moving is the worst. [I1] just moved on the weekend. So is it the stress of moving?*<br>Ah, no, it's just moving in general. Like having to pack your stuff. [11.F1]<br>*[I2] The disruption.*<br>Just want something that's stable, which is where I'm at now. [11.F1]<br>I'm the same. [11.F2]<br>*[I1] So you'd smoke because just the worry of having to move to a new place, move everything, and packing and unpacking.*<br>Yeah. [11.F1]<br>*[I1] Ok.*<br>But sometimes it's also the thought of not knowing where you're moving to. [11.F2]<br>*[I1] Ok, yeah, yeah.*

	And stuff like that. Cos I've moved houses quite a lot too, since I was about eighteen. And I felt like it did worry me sometimes cos I didn't know where I was go, so like her, I was couch hopping. I moved probably ten, fifteen times in the last couple of years. And right now, I'm in a transitional house, so I'm going to be moving again in a few weeks. Maybe, or it could be a month, or it could be two. I don't know.<br>So, it's always the, the fear of not knowing where I'm going to be able to set up a permanent house for me and the kids, yeah, cos we haven't had stability, we've moved, I've had the kids for about a year and a half now, and I've moved house three times. [11.F2]<br>*[I1] Wow, ok.*<br>And we've got to move again, and again, and it's just like, it stresses me out knowing that we can't unpack all our stuff, just because we know that we're going to be packing up again in a few months. And the kids, it's not fair for the kids. They don't get a chance to set up their rooms and they don't feel like they've got a stable home. Hmm. So that does stress me out as well. [11.F2]<br>*[I2] Are you feeling that stress too?*<br>*[I1] Do you feel it [M1]?*<br>Yeah. [11.M1]<br>*[I1] Yeah, that obviously would be an every day stressor.*<br>Well, I was basically homeless before I got guardianship of the kids. So I've had to find a stable house, I've had to rent private rental, pay an arm and a leg for a fricken week, just for rent. Just so I could get the kids, and then I've had to quit my job to look after the kids. And then I've had to move because I couldn't afford the rent. And I've had to move from that place to that place, and I'm just stressed. [11.F2]<br>*[I1] That's a huge combination of things, yeah. Yeah. But yeah, so having them everyday stresses, and having the instability, not being stable?*<br>Yeah. [11.F2]<br>*[I1] And having the, not have a choice of where you're living, being transient, everything else, yeah that would*<br>*[I2] That undercurrent of uncertainty, it's just always sitting there. You don't know what's going to change, when.*<br>*[I1] I hear your anger. I actually get a bit frustrated thinking about it. I hear you sis. Keep your head up.*<br>It is. [11.F2]<br>*[I1] It'll turn.*<br>That's why I need my smokes. [11.F2]
3.4 Nicotine addiction	Is there addiction in there? [7.M1]<br>*[I1] Addiction. Addicted.*<br>Yeah, that one. [7.F1]<br>*[I1] So, why have you chosen addiction, or addicted, brother?*<br>Um well, it comes under stress and, as well, because once you smoke, it, that's a way that it could make your stress go away. And once you have that, like… [7.M1]<br>Once you start smoking… [7.M2]<br>Yeah, you get heaps addicted to it because it takes away the stress. [7.M1]<br>*[I1] Yeah, so once you start, it's hard to stop?*<br>Yeah. [7.M1]
	Mine would probably be stress and addicted. Because you know, some people will try it once out of stress, you know, just to get rid of all your stress, and then after one, you become addicted, and you just keep going and going. [9.M3]
	I don't know. It could be like depressed. Like, feel like you harm yourself or whatever. And you need a smoke to calm you down or something. For example like yarndi. And then same thing, like if you're stressed out, like going through court, family members in hospital crook. Just want to smoke, try and relax yourself. And then addicted, since you started, you're just addicted. Ever since you started. [4.M1]
3.5 Family illness	If you have a family member that's sick and you see like, the pain and the stage that they're going through. You feel like upset because you don't want them to be like in that, in that stage, and it will help you not want to be like them, in the bed. [7.M1]
	[I1] Um sick family. So if you had family that was sick, and dying and saying affected through cigarettes, do you think that would turn you off smoking, seeing them pass away with time, and cancerous, and?<br>See there, I'm torn. Like yes it would turn me off smoking, cos obviously all the bad stuff instilled into your body and stuff, but it would also make me want to smoke, just because of the stress factor involved. So, my family member's dying, like there's nothing I can do, it's just it's gotta stress me out and it's my family's all stressing. Like, we're going to bury somebody, they're dying. Um, it would just like put my stress levels right up. So I'd be smoking because of the stress, but I'd also be thinking like I need to quit because look what it's done. You know what I mean? But I'm a bit torn with that. [11.F2]<br>*[I1] So, smoking, at the time as a calmer for yourself? So you'd smoke just to calm yourself*<br>Yeah [11.F2]<br>*[I1] Calm your stress?*<br>Yep. [11.F2]<br>*[I1] But at the same time you know that*<br>I know that it's doing more harm than good. Yeah. But just at that time, you feel like you just need to have a smoke just to catch your breath, catch your thoughts, just stop for a second. [11.F2]<br>*[I1] Ok. So that's going to smoking harms. You know what the smoking does to you, but at the same time you, your own*<br>At the same time, you're addicted and you can't just quit it like that. [11.F2]<br>*[I1] It's your calmer.*<br>Hmm. [11.F2]

3.6 Relationships	Yeah, cos it's like if you're stressing, you're always going to like have your family and friends there to support you, to not smoke and stuff. [7.F1]
	Um, just say if you feeling stressed your family can help you instead of smoking. Cos when people are stressed they smoke, or if other people are stressed, then their family can help them. [7.M2]
	And on this side, mine would probably be fitness and family. Cos if you have a strong family, they're behind you, you know, make you quit. Or you have a bad family that doesn't give a shit, you know, they just keep lending you, and fitness is also good to stay healthy. [9.M3]
Theme 4. Importance of individual empowerment and engagement for being smoke-free	
4.1 Disengagement, boredom and rejection of harms	Um, mine would be that friends smoke. I think it's also to do with trying to look cool. Um, I think you shouldn't have to wreck your body to try and look cool. [9.F1]
	[I1] Would you, would you encourage more people at the school, that don't smoke to say more? Like to say "don't smoke". Know what I mean? Like to encourage those that do.<br>Even the people who do smoke, like ask them why. Why are you doing that? [9.F1]<br>It's the quickest way to kill yourself because no way, [9.M1]<br>*[I1] Why do you say that, bud?*<br>Because some people will know that the cigarettes will actually kill them. Like eventually when they grow up. [9.M1]<br>*[I1] Right, so you're not talking suicide, we're just talking about longevity.*<br>Yeah, that's what they mean. They're all doing it because they think it won't happen to them, till like a couple of years. [9.M2]<br>*[I2] You saying that they're not really worried about the long term?*<br>Yeah, they're not worried about the long term, they worry about short term. [9.M2]<br>*[I2] Do you think overall people do know that it's harmful?*<br>Yeah. [9.M2]<br>*[I2] Ok. But they're just thinking it's ok for now?*<br>Yeah, exactly, they're like you know, "I'll quit after school". [9.M2]<br>*[I2] Quit after school?*<br>Hmm. When school finishes, they'll quit, but they just keep going. [9.M2]
4.2 Empowerment, engagement and self-efficacy	*[I1] Yeah? And do you, what, how do you think they started? Because of other people around them? So, influences. So just similarities to you, sis, where just being round the wrong crowd and that's sort of a tendency out this way.*<br>*[I2] Hmm, you've never been, never felt like that, that someone's trying to get you to start? That's good. Do you feel like you'd be able to say "it's not for me"?*<br>Yeah. I say no. [12.F2]<br>*[I1] And you will always be a non smoker? Yep. And you'll be a non smoker?*<br>Yeah, I got a future career I've got to focus on. Can't do that shit. [12.F2]
	At parties. Do it a lot at parties when they're drinking. They decide to smoke. I don't really like it. I just walk away from them. Cos I don't really like even seeing them doing that. [5.M1]<br>*[I1] And have they ever tried to*<br>Yeah, they try to ask me but I don't do it. [5.M1]<br>*[I1] Right. Good.*<br>*[I2] Do you feel ok about that?*<br>Yep. I like being different. And I like, you have to be your own person, you don't have to follow other people. I'd rather be a leader than a follower. [5.M1]
	[I1] And what does it make you think? Like do you want to be like them and smoke? Would you want to smoke like them?<br>Stay away from them. [3.M3]<br>*[I1] What turns you off then? What makes you think "oh, I don't want to do that"?*<br>Just your future. [3.M3]<br>Ask them what their dreams are. And then if they say whatever, then you say you're not going to get it by smoking. [3.M1]
	If I was with my friends and they pulled out a ciggy and started smoking I would like, I wouldn't be their friend. [7.M1]<br>I'd pull them up straight away. [7.M2]<br>*[I1] Oh yeah? What would you say?*<br>I'd just go that's not good for you. You shouldn't be smoking at this age. Yeah. In the long run, it's going to affect you, not anyone else but yourself. [7.M2]
	[I1] So they're still smoking now?<br>They are, yeah. I tell them to stop but you can't really force somebody to stop if they're not willing to stop, or not willing to take your help, your advice. [12.F1]

Designing a prevention program	
Objectives	*[I2] Yep. Um, for the high school kids, [M1], would it be preventing or help to quit, or everything?*<br>Yeah, probably everything. Yeah. [11.M1]<br>*[I1] What would you rather? Would you rather help to quit,*<br>Yeah. Help to quit. [11.M1]<br>*[I1] Or would you rather an awareness one? Help to quit. Um, would you rather, would you prefer one at school, so that you can stay at school?*<br>Yeah. One there. [11.M1]<br>*[I1] So a program at school?*<br>Hmm. [11.M1]
	[I2] Um, [F1], so your question was whether it was about preventing or getting people to stop. Do you think any of your friends are smoking enough that they're addicted, that they might need actual quit support? Like nicotine patches, or anything like that.<br>None of my friends, but there are some people at school who do smoke a lot. Like, even younger than me, they're in year ten, and they're smoking heaps of cigarettes. But you know, I think they just do it to look cool, cos they want to be cool type of people. Yeah. [10.F1]
Settings	*[RO] What are some things that you would like to see for your engagement maybe? Like what would make you engage in trying to quit smoking?*<br>Um, being able to go to the doctors. I know they say it to you now, but if you say no, I don't want to quit, or maybe I do feel like quitting. Maybe if they pushed it a bit more. [2.F2]<br>*[I2] Does your doctor ask you every time?*<br>Hmm, hmm. [2.F1]<br>*[I2] That's good.*<br>*[I1] Do they follow up though? Cos I know my doctors, they ask me every couple of months*<br>*[RO] So we did actually at [AMS] have a designated smoking worker, and she was following up, she was following up clients all the time, she was in the same office as us and she was ringing them at least once a week. But she actually left and it hasn't been filled since. But she was getting quite good success rates with that.*<br>*[I2] Do you appreciate that? When your doctor asks, and offers support, or is it "this is not what I'm coming here for, I've got other things I want to talk to you about".*<br>Yeah, I usually say that, "I'm not here to talk about quitting." [2.F2]<br>*[I1] Trying to push it aside.*
	Have a gala day on the oval or something, and like for people who smoke, like yarn up if you're depressed or stressing out. Just have a yarn, and try and give up the smokes. Don't put yourself in that situation to go down. You could, I don't know, your lungs collapse, whatever. Could be, could die. I don't know, just try to give it up and be strong. [4.M1]
Features	Be like, if they can get something like chemicals here, like I don't know. Like show like how smoking works. Like you know how they have that one lung and the other lung, and how you press it and how slow the air released out of the smoked lung? [7.M2]<br>*[I1] Yeah, yeah.*<br>Maybe like take some science stuff into class and stuff and show them how the smoke digests into like your lungs and stuff. [7.M2]<br>*[I1] So the harms of smoking, like a demonstration? Or comparison.*<br>So the physical so that they actually understand. [7.M2]<br>*[I2] Yeah, quite practical like you touch it, yeah, not just information. You get to see what it's actually doing to you.*<br>Some people just get bored listening to information, they'll just sit there, doze out. [7.M2]<br>Yeah, they do. Probably better to [7.F1]<br>*[I1] So, a hands on demonstration, yeah?*<br>Yeah. [7.M2]
	I reckon we should get some Elders that's like grew up smoking and quit. [9.M2]<br>Yeah, that's been around. [9.F1]<br>Sit around and tell you how much, tell the young 'uns you know, how it ruined them, and how they felt to inspire the little kids not to do it. [9.M2]<br>*[I1] So, so personal stories from those that experienced*<br>Smoking and grew up around it. [9.M2]
	I think the thing is, yeah, like personal reasons and that. Like I don't get like why have a program for smoking. Like some people are that stupid they're going to, they're going to think if you have some, a group of people in a room, and like tell them, you can't just tell them "stop smoking, stop smoking", and they're going to do it. So like unless they actually see the future, like what society will end up being like, like then yeah, ok. So, I don't know. It's just like I wouldn't want to run any program about smoking, cos people need to find out what smoking does themselves. [8.F2]<br>*[I2] That's a really good point. So we need to think about how we target non smokers and help them stay non smokers.*<br>Yeah. [8.F2]<br>*[I2] And you're saying talk to them about bigger picture and life in general.*<br>So, if I was to run a program, I'd get… [8.F2]<br>You don't want to miss out on anything. [8.F1]<br>I would literally get someone that's experienced for this. And I will, like I will make them know that smoking can like, have traumatic parts, it can do unhealthy things, and it can get you in the state where

	you don't want to be in trouble and all that. But other than that, I would not want to run any program about it. They will have to find it out themselves. [8.F2]
	[I2] I like the idea of "quit together" though. Like if there was something where there was a challenge, and you made like a team, or a group, is it then<br>Yeah, that sounds really good. I'd actually do that, yeah. [11.F2]<br>*[I2] What if you got like the women's group back, and part of that was yeah, checking in weekly, "how are you going", and?*<br>That will be cool. Because there are a lot of young mums around here, that smoke, well not just mums, single people too. Um, but yeah. Because there's been a bit of talk about wanting, I've asked a few girls about the women's group, and a lot of them want the women's group back. I do. I've been saying that for years. [11.F2]
	[I2] If there was like a structured program, where you guys moved around activities, but part of it was like a challenge to try and quit as a team, would that be appealing? Where you're doing it as a group big. You had different things that you were doing together as well, or?<br>Is there any initiative, like is there a T-shirt at the end of it or something? [11.F2]<br>*[I2] Oh, there's always a T-shirt, there's always merch.*<br>Yeah, there, there has to be something I can get out of it, eh? [11.F2]<br>*[I1] Well tell us, what would you like, what would youse like at the end?*<br>Well, like T-shirts are a very big deal around here. So if there was a deadly designed T-shirt at the end of it, that you get to keep, or maybe um, like a bag or something, that's covered in Koori designs, that'd, that'd be a real eye catcher. [11.F2]<br>*[I2] What if, as part of the Art Class, you guys did the design, and then it got made up into jumpers or T-shirts, or bags or something, and it was your design?*<br>That'd be cool. Yeah. Cos I know they done a health workshop over at um, over at the health centre. As part of my TAFE class, we had to go do health checks all day. And as an initiative for the community members to do, just to do the health check, cos they had a barbeque and all that. Um, whoever done a health check gets a NSW Blues Indigenous jersey. Yeah, but I missed out, I was so fuming. [11.F2]<br>Why would you want a shit team anyway? [11.F1]<br>You be quiet. But yeah, Indigenous jerseys they're always eye catchers for people. [11.F2]<br>*[I1] Ok, they're good suggestions, we haven't had those before.*<br>*[I2] Yeah, that's really good. And it'd be cool if you know*<br>*[I1] So locally designed*<br>Some local Koori design, yeah. [11.F2]<br>*[I1] So locally designed shirt, jumper, bags, yeah something like that.*<br>*[I2] Something that was just yours. Something that only the people who got to do the program, no one else could get it.*<br>Yeah. [11.F2]
Timing	I reckon long term would be better. [11.F2]<br>*[I1] And how do you*<br>You'd get better results. [11.F2]<br>*[I1] How often would you have it?*<br>I'd say weekly. [11.F2]<br>*[I1] Weekly?*<br>Yeah, once a week. [11.F2]<br>Yeah, weekly. [11.M1]<br>To run the program. I'd be up for that. [11.F2]<br>*[I1] You'd agree with that, sis?*<br>Yeah. [11.F1]<br>Yeah, once a week. For a couple of months. I'd say about nine, maybe. A bit long? [11.F2]<br>*[I2] I don't know, because of the…*<br>If you want to go like a quit smoking program, it's going to take time, and [11.F2]<br>*[I1] At least nine months, yeah?*<br>At least nine, yeah. Cos if I want to quit smoking, I know my behaviour's going to change and I want to her there with me, cos if she's quitting too, she's going to want me, and it's just going to be like, we know we've got another couple of months to go together, and like. So by the time we full on quit, you know, it'd be good to make new friends too. [11.F2]<br>*[I2] Yeah.*<br>Like there are other people who I don't really talk to, but I'm pretty sure if we like go through this program, and meet up, and we'll have something in common about quitting smoking. And it will be… [11.F2]<br>*[I2] And you'll be non-smokers together.*<br>Yeah. [11.F2]<br>*[I2] But yeah, I think the timeline is important, because if you stop and start, or you eventually becoming quit, you don't want to have like intensive program, and there's nothing to support you. You want, even if you do stop and start again, you're still in the program. You're still here. Keep coming.*<br>Just something constant over the next few weeks that we know is going to be in place. So like I'm not going to go cold turkey and then start smoking in a month or two, you know what I mean? [11.F2]
