# Supplementary material for: Don’t Follow the Smoke—Listening to the Tobacco Experiences and Attitudes of Urban Aboriginal Adolescents in the Study of Environment on Aboriginal Resilience and Child Health (SEARCH)
Source: Int J Environ Res Public Health. 2023 Mar 4;20(5):4587. doi: 10.3390/ijerph20054587 (PMC10002023; doi:10.3390/ijerph20054587)
Supplement: Supplementary file 1 [file ijerph-20-04587-s001.zip › S6 Large scale Figure 1 Concept map of card sorting activity.pdf]

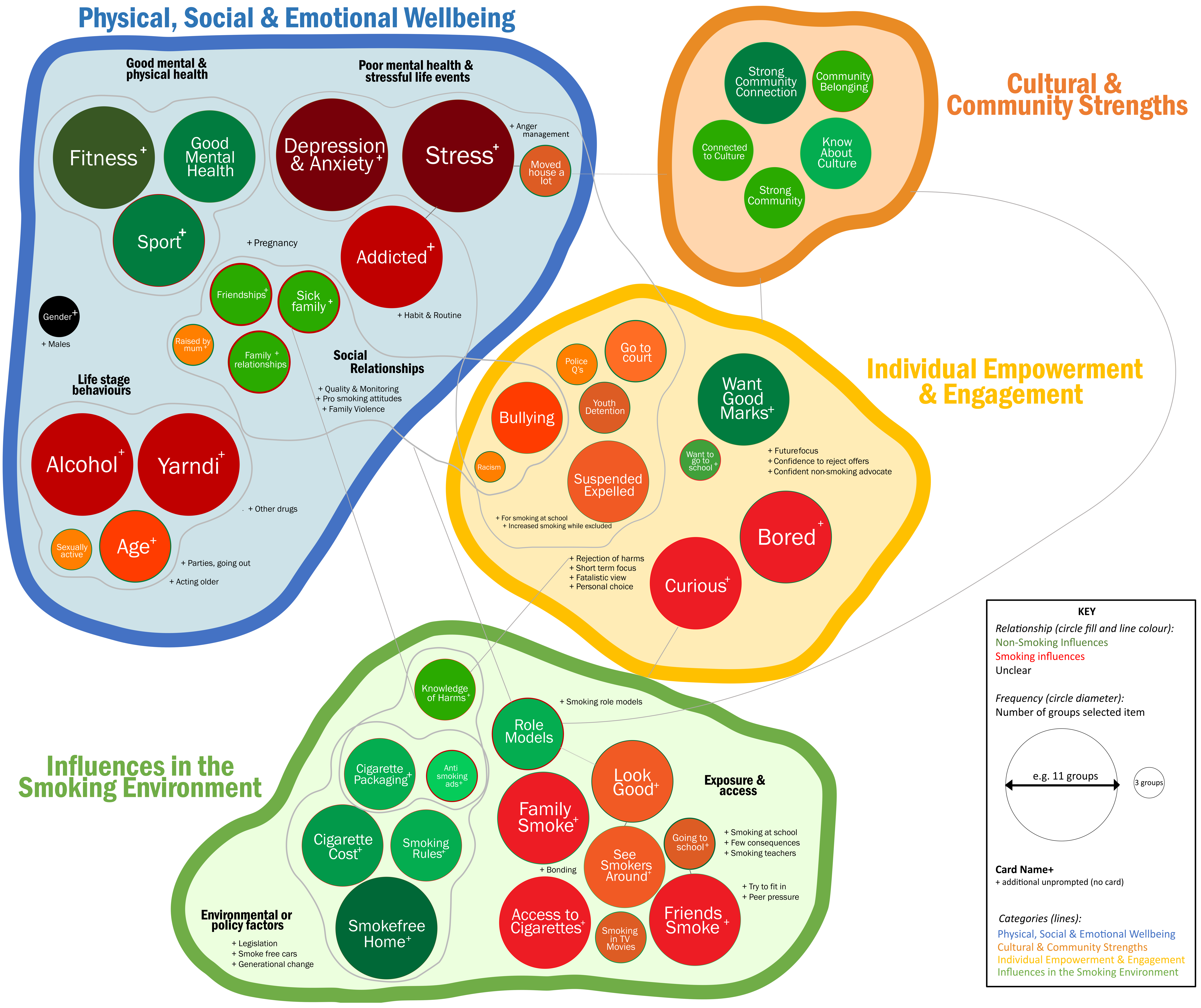
Physical, Social & Emotional Wellbeing
Good mental & physical health
Fitness+
Good Mental Health
Sport+
Poor mental health & stressful life events
Depression & Anxiety+
Stress+
+ Anger management
Moved house a lot
Addicted+
+ Habit & Routine
+ Pregnancy
Friendships+
Sick family+
Raised by mum+
Family+ relationships
Gender+
+ Males
Social Relationships
+ Quality & Monitoring
+ Pro smoking attitudes
+ Family Violence
Life stage behaviours
Alcohol+
Yarndi+
+ Other drugs
Sexually active
Age+
+ Parties, going out
+ Acting older
Cultural & Community Strengths
Strong Community Connection
Community Belonging
Connected to Culture
Know About Culture
Strong Community
Individual Empowerment & Engagement
Police Q's
Go to court
Bullying
Youth Detention
Racism
Suspended Expelled
+ For smoking at school
+ Increased smoking while excluded
Want Good Marks+
Want to go to school+
+ Futurefocus
+ Confidence to reject offers
+ Confident non-smoking advocate
Bored+
Curious+
+ Rejection of harms
+ Short term focus
+ Fatalistic view
+ Personal choice
Influences in the Smoking Environment
Knowledge of Harms+
Cigarette Packaging+
Anti smoking ads+
Cigarette Cost+
Smoking Rules+
Smokefree Home+
Environmental or policy factors
+ Legislation
+ Smoke free cars
+ Generational change
Role Models
+ Smoking role models
Look Good+
Family Smoke+
+ Bonding
See Smokers Around+
Going to school+
Exposure & access
+ Smoking at school
+ Few consequences
+ Smoking teachers
Access to Cigarettes+
Smoking in TV Movies
Friends Smoke+
+ Try to fit in
+ Peer pressure
KEY
Relationship (circle fill and line colour):
Non-Smoking Influences
Smoking influences
Unclear
Frequency (circle diameter):
Number of groups selected item
e.g. 11 groups
3 groups
Card Name+
+ additional unprompted (no card)
Categories (lines):
Physical, Social & Emotional Wellbeing
Cultural & Community Strengths
Individual Empowerment & Engagement
Influences in the Smoking Environment
